# A novel approach using *C. elegans* DNA damage-induced apoptosis to characterize the dynamics of uptake transporters for therapeutic drug discoveries

**DOI:** 10.1038/srep36026

**Published:** 2016-10-27

**Authors:** Arturo Papaluca, Dindial Ramotar

**Affiliations:** 1 Maisonneuve-Rosemont Hospital, Research Center, Université de Montréal, Department of Medicine, 5415 Boul. de l’ Assomption, Montréal, Québec H1T 2M4, Canada

## Abstract

Organic cation transporter (OCT) function is critical for cellular homeostasis. *C. elegans* lacking OCT-1 displays a shortened lifespan and increased susceptibility to oxidative stress. We show that these phenotypes can be rescued by downregulating the OCT-1 paralogue, OCT-2. Herein, we delineate a biochemical pathway in *C. elegans* where uptake of genotoxic chemotherapeutics such as doxorubicin and cisplatin, and subsequent DNA damage-induced apoptosis of germ cells, are dependent exclusively upon OCT-2. We characterized OCT-2 as the main uptake transporter for doxorubicin, as well as a number of other therapeutic agents and chemical compounds, some identified through ligand-protein docking analyses. We provide insights into the conserved features of the structure and function and gene regulation of *oct-1* and *oct-2* in distinct tissues of *C. elegans*. Importantly, our innovative approach of exploiting *C. elegans* uptake transporters in combination with defective DNA repair pathways will have broad applications in medicinal chemistry.

The nematode *Caenorhabditis elegans* has a plethora of advantages as a useful *in vivo* model system^1^. Indeed, this organism exhibits a broad array of phenotypes that can be easily monitored for changes in various genetic/physiological pathways. For example, it can be utilized to understand the roles of Organic Cation Transporters (OCTs) in the uptake of therapeutic substrates^2^. In fact, evidence from mammalian systems dictates that OCTs mediate the uptake of chemotherapeutic drugs such as oxaliplatin and daunorubicin^3^,^4^. Hence, elucidating the molecular underpinnings of OCTs and consequent development of tools to modulate their transport activity *in vivo* are expected to improve chemotherapeutic outcome.

In *C. elegans*, little is known about the roles of OCTs and their affinity towards distinct substrates. OCT-1 was the first uptake transporter characterized from *C. elegans* and when expressed in mammalian cells was shown to mediate the transport of the organic cation tetraethylammonium, a prototypical substrate used for classifying OCTs^5^. *C. elegans* deleted for *oct-1* exhibits a shortened lifespan and increased susceptibility to oxidative stress, which led to the proposition that OCT-1 facilitates the import of antioxidants required to protect *oct-1* mutant animals from oxidative stress^2^. However, uptake of ergothioneine, the purported antioxidant substrate of OCT-1, was not reduced in *oct-1* mutant animals as compared to the parent^2^. Therefore, it seems plausible that an alternative explanation could account for the *oct-1* mutant animal phenotypes.

Recently, we documented that the expression of *C. elegans* OCT-1 can restore uptake of the chemotherapeutic drug doxorubicin into *Saccharomyces cerevisiae* cells lacking the regulator Agp2, an amino acid transporter that when deleted blocked the expression of several target genes including the polyamine transporters Dur3 and Sam3^6^. No further studies were done to determine whether OCT-1 substituted for the regulatory function of Agp2 or directly for the roles of Dur3 and Sam3, as both of these transporters also mediate the transport of doxorubicin^6^. Furthermore, it remained unknown whether OCT-1 might mediate the transport of doxorubicin into *C. elegans*. Besides OCT-1, *C. elegans* possesses another related member of the SLC22 organic cation transporter family, i.e., OCT-2. OCT-2 shares 22.56% identity with OCT-1, but differs in having an extended N-terminal of 172 amino acid residues that is unrelated to OCT-1. To date, no studies have assigned a structural and functional role to the putative *C. elegans* OCT-2 transporter.

During the last decade, *C. elegans* has become instrumental in several drug discovery programs^1^,^7^. However, in many high-throughput screens performed so far to identify novel small molecules, e.g., those that act as antimicrobials, extend lifespan, inhibit oxidative stress or prevent multidrug resistance, the yield of bioactive compounds is typically in the range of 0.03% to less than 1%^1^,^8^. It is possible that the recovery rate could be higher if there is greater influx of the molecules. High-throughput screens at higher initial concentrations of the small molecules may alleviate this issue, but could be cost prohibitive. As such, we propose that characterization of the function and substrate specificities of uptake transporters in *C. elegans* will be advantageous towards improving the strategies employed to identify novel bioactive molecules.

In this study, we report a number of novel findings regarding the OCT-1 and OCT-2 transporters of *C. elegans*. We show for the first time that (i) unlike the downregulation of *oct-1*, depletion of *oct-2* did not affect the lifespan of the animals and, instead, rescues the shortened lifespan of *oct-1* deletion animals, (ii) *oct-1* downregulation leads to *oct-*2 upregulation, which in turn mediates uptake of toxic environmental compounds and chemotherapeutic drugs, (iii) upregulation of OCT-2 increases uptake of prooxidants, as judged by the activation of the oxidative stress response reporter GST-4::GFP, leading to germ cell death, as well as to damages to other tissues that could account for the shortened lifespan of *oct-1* deletion animals, (iv) oct-2 upregulation mediates the accumulation of clinically relevant genotoxic anticancer drugs that sensitizes DNA repair deficient animals to germ cell death and diminishes their survival, and (v) ligand-protein docking analysis can be exploited to define substrates such as DNA damaging agents that tightly bind to OCT-2 and which can be validated by suitable readouts. Our findings represent a robust OCT-based strategy to screen a plethora of new therapeutic drugs useful for treating human illnesses, and provide crucial information for rapid recognition of their pharmaceutical benefits and adverse effects.

## Results

### oct-2 deficiency rescues the shortened lifespan of oct-1 deletion mutants

**Figure 1:**
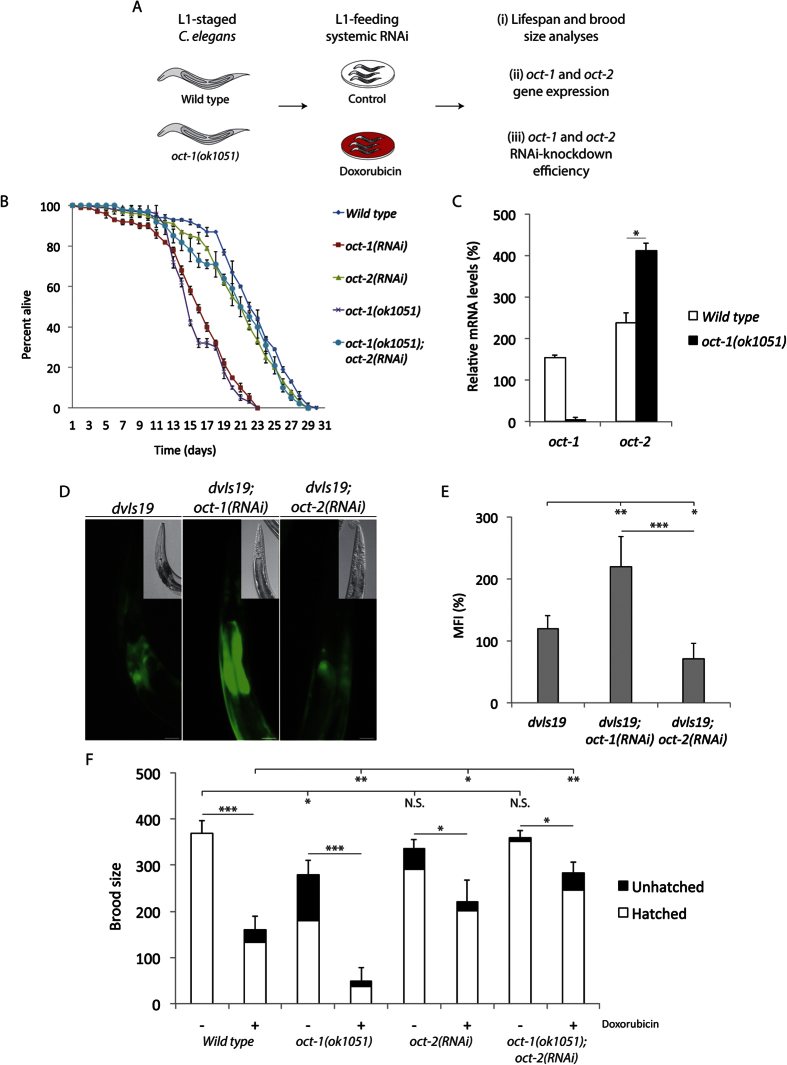
Upregulation of *oct-2* gene expression shortens the lifespan and increases the sensitivity of *C. elegans* towards doxorubicin. (**A**) Scheme of the experimental design and the readouts analyses under different conditions. (**B**) Lifespan analysis of the indicated genotypes. L1-staged animals (*n* = 100) were fed the control and the indicated RNAi under normal growth conditions and lifespan was blindly assessed starting from young adult animals. The mean lifespan of two independent experiments is depicted. (**C**) The relative gene expression of the *oct-1* and *oct-2* transcripts in the wild type and the *oct-1(ok1051*) deletion mutant animals was measured and corrected on actin as an internal control. Synchronized young adult animals were collected and mRNA levels were assessed by qRT-PCR. Data shown represent the average ± s.d. and student T-test *P < 0.05 from a 60 × 15 mm petri dish of animals (*n* ~ 1000) pooled from two independent experiments. (**D**) Representative images of the oxidative stress-inducible GST-4::GFP reporter and DIC (higher right) of the indicated genotypes. All images were maximum projections of the whole *dvsI19* strain, but cropped from the pharynx to the primary section of the intestine for representation. (**E**) Increased GFP intensity depicting uptake of prooxidants. The GST-4::GFP activation was measured with Fluoroskan Ascent Microplate reader set at λ^ex^544 nm–λ^em^590 nm. The mean fluorescence intensity (MFI) is represented as percentage corrected on wild type. (**F**) Brood size analysis of the indicated animals under standard conditions (no treatment) and upon exposure to 100 μM doxorubicin. The data are mean ± S.D. No treatment: Wild type = 369.5 ± 27 (*n* = 17), *oct-1(ok1051*) = 280 ± 29 (*n* = 23), *oct-2(RNAi*) = 335.5 ± 20.5 (*n* = 20), *oct-1(ok1051*)*; oct-2(RNAi*) = 360.5 ± 14.8 (*n* = 21). Doxorubicin treatment (100 μM): Wild type = 170 ± 29.1 (*n* = 24), *oct-1(ok1051*) = 58 ± 29.2 (*n* = 19), *oct-2(RNAi*) = 220.5 ± 47 (*n* = 21), *oct-1(ok1051*)*; oct-2(RNAi*) = 283 ± 23.1 (*n* = 27). Error bars represent the S.D. Unpaired two-tail t-test *P < 0.03; **P < 0.01; ***P < 0.0005 were considered to be statistically significant. N.S. = Non Significant.

It has been postulated that *C. elegans* mutants deleted for *oct-1* are defective in uptake of antioxidants and, as a consequence, exhibit shortened lifespan and increased susceptibility to oxidative stress^2^. However, the fact that *oct-1* mutants show no defect in the uptake of the key antioxidant ergothioneine is inconsistent with this hypothesis. As such, we postulate that *oct-1* gene deletion could instead lead to increased uptake of prooxidants from the environment if the loss of OCT-1 activates expression of a related transporter. This notion is based on the fact that *OCT1* knockout mice manifest upregulation of two related transporter genes, *OCT2* and *OCT3*^9^. We therefore performed a homology search using *C. elegans* OCT-1 as a query for protein sequences in the *C. elegans* Wormbase. This analysis revealed a second *C. elegans* member of the organic cation transporter family SLC22, i.e., OCT-2, which shares 22.56% identity with OCT-1 (Figure S1). The predicted protein sequence of OCT-2 indicated that it possesses an extended N-terminal of 172 amino acid residues (Figure S1), suggesting that it is structurally distinct from OCT-1. To date, there is no previous report assigning a functional role to this putative transporter OCT-2 in *C. elegans*. We set out to characterize the molecular function of OCT-2 by first evaluating whether its RNA-interference (RNAi)-driven depletion might influence lifespan of *C. elegans*. In this experiment, L1-staged wild type animals were systematically fed bacteria harbouring the HT115 RNAi vector targeting *oct-2* to measure adult lifespan, using *oct-1* downregulation or the *oct-1* gene deletion mutant *oct-1(ok1051*) for comparison (Fig. 1A)^10^. As expected, *oct-1(RNAi*) or *oct-1* deletion mutants exhibited a shortened lifespan compared to wild type (Fig. 1B)^2^. In contrast, *oct-2(RNAi*) animals displayed a nearly normal lifespan (Fig. 1B). Quantitative real-time PCR (qRT-PCR) was used to ensure that *oct-1* and *oct-2* expression were indeed downregulated by the RNAi-driven approach (Figure S2A), and that this did not interfere with the expression of another transporter gene, namely *pes-23* (Figure S2B), indicating that RNAi did not have off-target effects. Surprisingly, when *oct-2* was downregulated in the *oct-1* deletion mutant *oct-1(ok1051*), the resulting *oct-1(ok1051*)*; oct-2(RNAi*) animals exhibited prolonged lifespan as compared to the *oct-1(ok1051*) mutants alone and approaching that of wild type worms (Fig. 1B). Thus, it would appear that the *oct-1* mutant phenotype is dependent on OCT-2 function. A simple interpretation is that depletion of OCT-1 may cause the upregulation of *oct-2* expression, such that OCT-2 mediates the uptake of toxic compounds that affect survival.

A gene expression analysis dataset available from the Wormbase for both *oct-1* and *oct-2*^11^, revealed that *oct-2* expression is normally higher than *oct-1* across *C. elegans* developmental stages (i.e. from the first larval stage (L1) to the fourth larval stage (L4)), as well as in the hermaphrodite gonads (Figure S3). We examined whether downregulation of *oct-1* would alter *oct-2* expression levels by comparing the *oct-1* and *oct-2* gene expression in the wild type to that of the *oct-1(ok1051*) mutant. We found in the latter that *oct-2* gene expression was significantly augmented, while the *act-1* mRNA levels, used as a control, were unchanged (Fig. 1C). This finding further supports the notion that the effects of *oct-1* depletion on the lifespan of the worms are attributable to *oct-2* upregulation.

### The SKN-1 target GST-4::GFP is upregulated by oct-1(RNAi), and blocked by oct-2(RNAi)

We examined whether the elevated levels of oxidative damage reported for the *oct-1* deletion mutant is dependent on OCT-2 function^2^. To test this, we used a reporter strain *dvls19* (GST-4::GFP) in which the promoter of the *gst-4* gene (encoding glutathione *S*-transferase 4) is fused to GFP. GST-4 is a target for the conserved SKN-1/Nrf2 transcriptional activator that plays a role in the defense against oxidative stress^12^,^13^. The *dvls19* strain showed a basal level of GST-4::GFP expression in the intestine of a representative animal (Fig. 1D), which was upregulated following RNAi downregulation of *oct-1* (Fig. 1D). In contrast, this *dvls19* strain showed lower than basal levels of GST-4::GFP expression when *oct-2* was downregulated by RNAi (Fig. 1D) and quantified by plate assay (Fig. 1E). These findings are consistent with a model whereby *oct-2* upregulation, *via oct-1* deletion, allows entry of toxic compounds such as prooxidants into *C. elegans* causing oxidative stress and leading to a shortened lifespan.

### OCT-2, and not OCT-1, mediates the genotoxic effects of the anticancer drug doxorubicin

To determine whether OCT-1 regulation of OCT-2 would be involved in the differential uptake of toxic compounds, we treated worms with the chemotherapeutic drug doxorubicin at concentrations that did not lead to developmental arrest^14^, and monitored the survival of the animals by scoring brood size. Doxorubicin uptake depends upon cationic transporters in *Saccharomyces cerevisiae*^6^ and mammalian cells^4^, and has been shown to trigger germ cell apoptosis in *C. elegans*^14^. L1stage wild type animals treated with doxorubicin showed ~55% decrease in brood size as compared to the untreated animals (Fig. 1F). In contrast, the *oct-1(ok1051*) mutants displayed a significant level of unhatched or dead embryos, and when treated with doxorubicin showed a sharp decrease (nearly 80%) in brood size (Fig. 1F). While these observations were unexpected as the loss of the uptake transporter should cause drug resistance, we reasoned that the enhanced doxorubicin sensitivity of the *oct-1(ok1051*) mutant animals can be explained by an increase uptake of the drug due to the upregulation of *oct-2*. We therefore examined the sensitivity of worms depleted for *oct-2* following exposure to doxorubicin. *oct-2(RNAi*) caused wild type animals to become less sensitive to doxorubicin with only 27% decrease in brood size, as compared to 55% for the control RNAi (Fig. 1F). Importantly, RNAi-driven depletion of *oct-2* in the *oct-1(ok1051*) mutant partially suppressed embryonic arrest and abolished the hypersensitivity of these animals towards doxorubicin showing less than a 20% reduction in brood size compared to 80% in *oct-1(ok1051*) mutant RNAi control (Fig. 1F). *oct-2(RNAi*) did not completely block doxorubicin toxicity on brood size of the *oct-1(ok1051*) mutant, perhaps reflecting residual *oct-2* expression. Also since this mutant was deleted for the *oct-1* gene, a direct role for OCT-1 in doxorubicin uptake is excluded (Fig. 1F). Collectively, these observations strongly support the notion that OCT-2 has a predominant role over OCT-1 in uptake of doxorubicin.

### OCT-2 allows the accumulation of doxorubicin in C. elegans tissues

**Figure 2:**
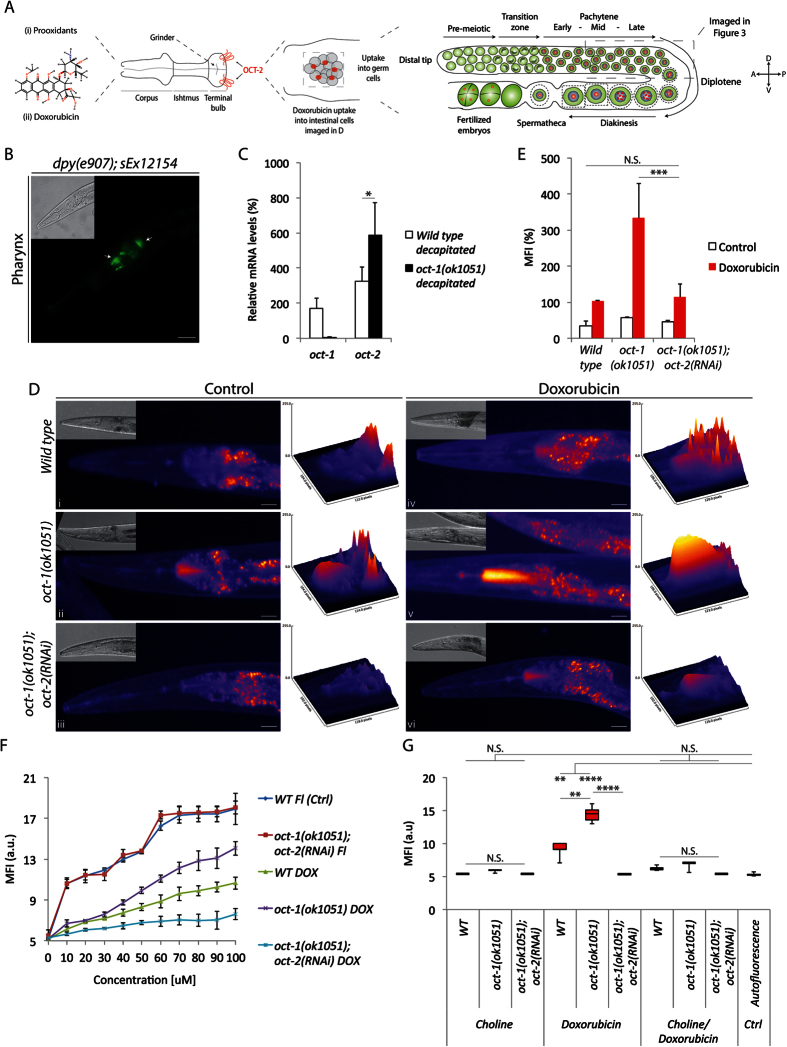
Doxorubicin transport into the pharynx is stimulated by *oct-2* upregulation and efficiently competed by choline. (**A**) Model suggesting OCT-2 localization at the terminal bulb of the pharynx, the fluorescence (red dots) where the drug is detected, i.e., the posterior side of the pharynx and the initial part of the intestine (as imaged in **F**), and the region of germ cells analysed for apoptotic corpses in the gonad (see below). (**B**) DIC (upper left) and fluorescence image of *dpy-5(e907*)*; sEx12154* [OCT-1::GFP] genotype depicting OCT-1 localization in the pharynx (arrows in **B**). Enlargement of the pharynx is represented by a scale bar = 7 μm. (**C**) Relative gene expression of *oct-1* and *oct-2* transcripts measured in severed heads (*n* = 300) collected from wild type and *oct-1(ok1051*) animals and corrected to actin and *pmp-3*. (**D**) Representative ‘fire’ look-up images of the pharynx from untreated (control) and doxorubicin treated animals. The respective DIC images are shown in the upper left corner of each panel. Images to the right of each pharynx depict a 3D representation of the doxorubicin (100 μM) treatment signal intensity for the indicated genotypes. Data is representative of experiments performed in duplicates (*n* = 20). Enlargement of the pharynx is represented by a scale bar = 10 μm. Fluorescence posterior to the pharynx is autofluorescence detected from the intestine. (**E**) Quantification of doxorubicin uptake using a Fluoroskan plate reader. The mean fluorescence intensity (MFI) is represented as percentage corrected to wild type. White bars denote untreated animals where similar autofluorescence was detected in all genotypes. (**F**) *oct-2(RNAi*)diminishes the concentration-dependent uptake of doxorubicin (DOX) and not fluorescein (Fl) into the pharynx of *oct-1(ok1051*) mutant animals. (**G**) Comparison of doxorubicin uptake in the absence and presence of equimolar amounts of choline. Box and whisker plots represent duplicates (*n* = 10) of signal intensity measured from both compounds in the pharynx. Data is represented as ± S.D Student T-test ± S.D Student T-test *P < 0.05; **P < 0.01; ***P < 0.001; ****P < 0.0001 and N.S. = Non Significant.

Based on the above findings, we examined whether OCT-2 might allow the accumulation of doxorubicin in *C. elegans* tissues. As target we chose the pharynx, a relatively large muscular organ that allow food consumption (Fig. 2A). We found that OCT-1 was localized at the anterior and posterior sides of the terminal bulb of the pharynx by imaging the *dpy-5(e907*)*; sEx12154* strain featuring an *oct-1::GFP* fusion (Fig. 2B), thereby validating this organ for uptake studies. Since no similar *oct-2::GFP* fusion has yet been constructed, we assessed the pharynx for *oct-2* expression by measuring RNA levels in the heads, severed just posterior to the pharynx, of 300 each of wild type and *oct-1(ok1051*) animals. The results reveal that *oct-2* is expressed at higher levels than *oct-1* in the pharynx of the wild type animals and that its expression was stimulated nearly 2-fold in *oct-1(ok1051*) animals as compared to the wild type (Fig. 2C), suggesting that *oct-2* is inducible in the pharynx.

We took advantage of the physical property of doxorubicin, which emits fluorescence at wavelengths of λ^ex^470 nm–λ^em^585 nm, as a convenient means to monitor its uptake through OCT-1 and OCT-2 *in situ* by imaging the pharynx. Of note, *C. elegans* tissues have low levels of autofluorescence particularly at the posterior terminal bulb of the pharynx (Fig. 2D[i]) where most substances accumulate prior to consumption^15^. However, following exposure of wild type animals to doxorubicin, the emitted fluorescence was greater than background autofluorescence confirming consumption of the drug by the animals (Fig. 2D[iv]). Strikingly, the *oct-1(ok1051*) mutant did not reduce the fluorescence intensity of doxorubicin in the pharynx, instead depicting a markedly stimulated intensity (Fig. 2D[v]). To test whether the stimulated uptake of doxorubicin in the *oct-1(ok1051*) mutant could result from upregulation of *oct-2*, we downregulated *oct-2* expression in this mutant. Under this condition, extremely low levels of doxorubicin accumulated in the *oct-1(ok1051*)*; oct-2(RNAi*) animals (Fig. 2D[vi]). It is noteworthy that the deletion mutant *eat-2(ad453*), i.e., with a slow pumping pharynx^16^, also showed OCT-2-dependent uptake of doxorubicin, excluding the possibility that this transporter functions only when there is a surplus of nutrients (Figure S4).

Doxorubicin uptake in the pharynx of the *oct-1(ok1051*) strains was independently confirmed using another assay employing the Fluoroskan instrument, which measures the fluorescence intensity of the drug (Fig. 2E). Using this approach, *oct-1(ok1051*) animals showed a concentration-dependent uptake over a range (10–100 μM) of doxorubicin into the pharynx, which was blocked by RNAi-driven depletion of *oct-2* (Fig. 2F). We tested whether uptake of another fluorescence compound, fluorescein which is anionic in nature, would be similarly dependent upon OCT-2. While fluorescein showed a concentration dependent uptake, ranging from 1 to 100 μM, into the pharynx of the *oct-1(ok1051*) mutant, it was not affected by *oct-2* downregulation in *oct-1(ok1051*)*; oct-2(RNAi*) animals (Fig. 2F). These findings suggest that OCT-2 may recognize differences in charges and display specificity in the uptake of substrates into *C. elegans* tissues.

### OCT-2-mediated uptake of doxorubicin is blocked by choline in the pharynx

Human OCT1 and OCT2 were shown to transport other cationic molecules such as choline^17^. Thus if choline is also a substrate of *C. elegans* OCT-2 we would expect it to compete for doxorubicin uptake into the pharynx. Choline has the physical property of emitting fluorescence, but at lower wavelengths of λ^ex^290 nm–λ^em^345 nm and is undetectable at the wavelength index used for monitoring doxorubicin uptake. Treatment of the *oct-1(ok1051*) animal with doxorubicin in the presence of equimolar amounts of choline (50 and 100 μM), impeded uptake of doxorubicin into the pharynx (Fig. 2G), clearly indicating that OCT-2 has the ability to recognize and compete for other cationic compounds. Therefore, we predict that the competition for doxorubicin uptake can be readily exploited as an assay to determine whether a putative ligand can serve as a substrate for OCT-2.

### The oct-1(ok1051) mutant animals display increased spontaneous and drug-induced germ cell death that is suppressed by oct-2 downregulation

**Figure 3:**
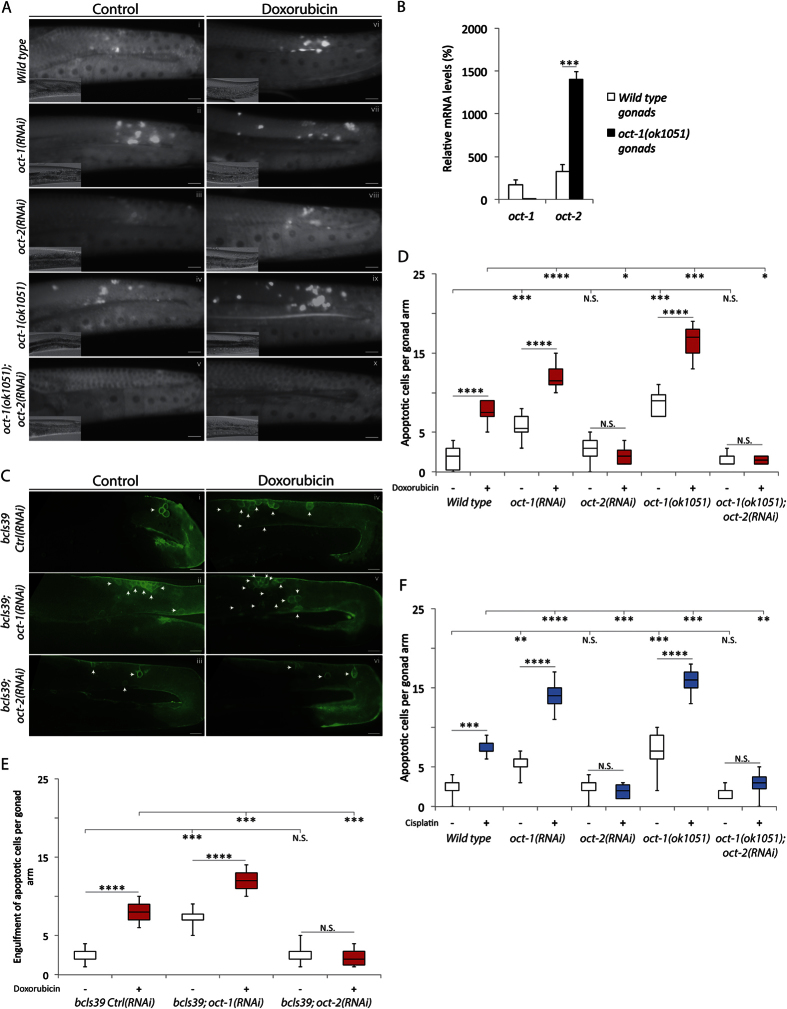
OCT-2-mediated transport of genotoxic compounds induce apoptotic cell death of meiotic germ cells in *C. elegans*. (**A**) Representative images of acridine orange-stained and DIC (lower left) of control and doxorubicin-induced apoptotic cell corpses from the indicated genotypes. Apoptotic cell corpses were identified as bright spots correlating with raised-bottom-like refractive corpses shown on DIC images. Posterior is right and dorsal is top. Scale bar = 15 μm. (**B**) Relative gene expression of *oct-1* and *oct-2* transcripts measured in dissected gonads (*n* = 100) collected from wild type and *oct-2(ok1051*) animals and corrected to actin and *pmp-3* (see experimental procedures). (**C**) Representative images of control and doxorubicin-induced *bcls39* [CED-1::GFP] clusters around apoptotic cell corpses indicated by white arrows. Data showing that OCT-2 mediated transport of compounds signal the apoptotic pathway in germ cells. Scale bar = 10 μm. (**D**) Box and whisker plots depicting quantification of apoptotic cell corpses from untreated and doxorubicin treated worms and showing maximum, minimum, upper & lower quartiles, and sample median. Statistical significance bars represent results of Mann-Whitney U-test of mean difference (*P < 0.05; **P < 0.01; ***P < 0.001 and ****P < 0.0001) computed from three independent experiments (*n* = 30). L1-stage animals were treated with doxorubicin (100 μM), and apoptotic cell corpses were analysed in young adult staged animals. Control is depicted as white and doxorubicin as red boxes. (**E**) Quantification of *bcls39* engulfment of apoptotic cell corpses in the indicated genotypes without and with doxorubicin treatment. The results are presented as shown in (**D**). (**F**) Box and whisker plots depicting quantification of apoptotic cell corpses from untreated and cisplatin treated worms and quantified as in (**D**).

Analogous to many stem cell systems, *C. elegans* has a self-renewing germ cell population derived from a cellular niche located at the distal tip (see Fig. 2A)^18^. These germ cells progress through distinct stages of differentiation and must faithfully maintain the genome. They are very sensitive to genotoxic compounds and respond by using conserved DNA repair mechanisms to maintain genomic stability^19^. Germ cells with excessive DNA damage undergo apoptosis and are unable to form viable embryos^18^,^20^. We chose to monitor germ cell apoptosis as an experimental system to determine whether OCT-2 would be involved in the uptake of genotoxic anticancer drugs. This approach has the advantage of allowing assessment of the uptake of genotoxic anticancer drugs that do not emit fluorescence or are unavailable in radioactively labeled form. To monitor apoptotic germ cells, we first quantified *in vivo* germ cell corpses in the proximal zone of the gonad arm (see Fig. 2A) by utilizing differential interference contrast (DIC) microscopy and staining with the DNA dye acridine orange^21^. In wild type animals, one to four apoptotic cells were detected (Fig. 3A[i]), as previously reported^22^. In contrast, the *oct-1(RNAi*) or the *oct-1(ok1051*) mutants depicted an average of five to eight apoptotic cells (Fig. 3A[ii,iv]). Analysis of gene expression in the gonads from 100 each of dissected wild type and *oct-1(ok1051*) animals revealed that *oct-1* gene deletion greatly stimulated the expression of *oct-2* mRNA (Fig. 3B). Thus, the two-fold increase of apoptotic cells in the gonads of the *oct-1(ok1051*) animals may result from import of prooxidants, as assessed by activation of GST-4::GFP, that can damage the genome of the germ cells leading to embryos with hatching defects (Fig. 1F). Unlike the *oct-1(RNAi*), the *oct-2(RNAi*) animals displayed nearly the same average number of apoptotic cells as the wild type (Fig. 3A[iii vs. i]). Interestingly, RNAi-driven depletion of *oct-2* in the *oct-1(ok1051*) mutant sharply reduced germ cell death, which was undetectable in some animals (Fig. 3A[v]).

To confirm that the acridine orange-stained germ cells are undergoing apoptosis, as well as to avoid uptake differences of this dye, we evaluated a downstream step of the apoptotic pathway, i.e., engulfment of apoptotic cells by the CED-1 protein to signal phagocytic degradation^23^,^24^. We employed an imaging strategy that utilizes the *bcls39* strain which carries CED-1::GFP as a reporter of engulfed apoptotic cells^25^. The *bcls39* strain with control RNAi showed engulfment of 1 to 3 physiological apoptotic cells (Fig. 3C[i]), whereas downregulation of *oct-1* engendered increased engulfment recapitulating the enhanced germ cell apoptosis observed in the *oct-1(ok1051*) mutant (Fig. 3A[iv] vs. C[ii]). As expected, downregulation of *oct-2* in the *bcls39; oct-2(RNAi*) background displayed control levels of engulfment (Fig. 3C[iii]). Taken together, we propose that OCT-2 possesses the ability to transport toxic compounds such as prooxidants that cause germ cell death. These prooxidants are likely to cause damages to various tissues and may therefore account for reduced lifespan observed in the *oct-1(RNAi*) or *oct-1(ok1051*) animals (Fig. 1B).

We examined whether the OCT-2 dependent accumulation of doxorubicin would lead to the stimulation of germ cell death. Treatment of wild type worms with doxorubicin elevated the levels of apoptotic cell corpses as visualized by both acridine orange staining (Fig. 3A[vi]) and engulfment of the cells (Fig. 3C[iv]) (and quantified as in Fig. 3D,E, respectively). *oct-1(RNAi*) or the *oct-1* deletion mutants treated with doxorubicin showed substantially higher levels of acridine orange-stained apoptotic cells (Fig. 3A [vii,ix] and quantified as in Fig. 3D), which paralleled increased CED-1::GFP engulfment of the cells as compared to the wild type (Fig. 3C[v vs. iv] and quantified as in Fig. 3E). In contrast, far fewer engulfed apoptotic corpses appeared in doxorubicin exposed animals downregulated for *oct-2* (Fig. 3C[vi]). As expected based on acridine orange staining and engulfment of apoptotic cells, mutants deleted for *cep-1*, *egl-1*, *ced-9*, *ced-4* or *ced-3*, which manifest defects in the apoptotic pathway did not show enhanced apoptotic corpses (Figure S5[ii–vi], respectively), i.e., unlike wild type animals upregulated for OCT-2 and treated with doxorubicin (Figure S5[i]). Several conclusions can be derived from these observations: (i) OCT-2 has a predominant role over OCT-1 in the uptake of doxorubicin, (ii) doxorubicin uptake leads to induced germ cell death that correlates with decrease survival, and (iii) both the drug uptake and the induced-germ cell death are OCT-2 dependent.

### Cisplatin-induced germ cell death requires OCT-2 function

We assessed whether germ cell death in our experimental model can be used to monitor the uptake of genotoxic drugs that are not readily available as either fluorescently- or radioactively-labeled form. Since human OCT1 has been shown to transport members of the platinum family of anticancer drugs that act by creating intra- and inter-strand DNA cross-links^26^, we tested the role of *C. elegans* OCT-1 and OCT-2 in the uptake of cisplatin using germ cell death as a reporter. We monitored germ cell apoptosis in the absence and presence of cisplatin in the following four genotypes: wild type, *oct-1(ok1051*)*, oct-2(RNAi*) and *oct-1(ok1051*)*; oct-2(RNAi*). Cisplatin induced an increased level of germ cell death in the wild type, which was further greatly stimulated in the *oct-1(ok1051*) mutants (Fig. 3F). Downregulation of *oct-2* in the *oct-1(ok1051*) mutants prevented cisplatin-induced germ cell death (Fig. 3F). These observations strongly indicate that germ cell death induced by cisplatin primarily depends upon its uptake via OCT-2. We noted that the DNA damaging agent methyl methanesulfonate (MMS), which alkylates DNA bases resulting in both DNA-single and -double strand breaks, induced germ cell death, but independently of OCT-1 and OCT-2 function (Figure S6A). Likewise, γ-rays that create multiple DNA lesions also induced germ cell death (Figure S6B) independently of these transporters (Figure S6C). Thus, the transporter function of OCT-2 is not directly involved in the process of germ cell death induced by the DNA damaging agents.

### OCT-2-dependent transport of doxorubicin or cisplatin stimulates germ cell death in C. elegans mutants defective in DNA repair

We next systematically examined whether mutants defective in major DNA repair pathways would show OCT-2 dependent sensitization of DNA damage-induced germ cell death. As shown in Figure S7B, the *rad-51* deletion mutant, *rad-51(ok2218*), lacking the RAD-51 protein needed for DNA strand invasion during homologous recombination (HR)-dependent double strand break repair, exhibited higher endogenous levels of apoptotic cell death due to spontaneous unrepaired meiotic breaks^27^ as compared to wild type (Figure S7A). Treatment of the *rad-51(ok2218*) mutant with doxorubicin greatly stimulated apoptosis, which was further induced upon downregulation of *oct-1* in the *rad-51(ok2218*)*; oct-1(RNAi*) genotype (Figure S7C). Consistent with this data, *oct-2* gene expression was indeed upregulated in the *rad-51(ok2218*)*; oct-1(RNAi*) genetic background (Figure S7J). In contrast, depletion of *oct-2* by RNAi in *rad-51(ok2218*) animals suppressed the high level of apoptotic cells observed in this mutant upon exposure to doxorubicin (Figure S7C). These data indicate that upregulation of *oct-2* burdens the *rad-51(ok2218*) animals with doxorubicin-induced DNA lesions leading to enhanced germ cell death.

We also found that *apn-1(tm6691*) animals lacking the key enzyme APN-1, required for removing a variety of DNA lesions including oxidized bases via the base-excision DNA repair (BER) pathway^28^, showed enhance germ cell death by doxorubicin when *oct-2* expression was stimulated (Figure S7E,K). This induced apoptosis was strongly attenuated following depletion of *oct-2* by RNAi (Figure S7E). These data suggest that BER in *C. elegans* is also involved in processing doxorubicin-induced oxidative DNA lesions^29^ but, more importantly, OCT-2 controls the toxicity of the drug in *apn-1(tm6691*) mutant animals.

Unlike doxorubicin, cisplatin damages the DNA by creating DNA cross-links most of which are processed by the nucleotide excision repair (NER) and DNA mismatch repair (MMR) pathways^30^. We set out to investigate whether *oct-2* expression levels modulate cisplatin-induced germ cell death in mutants defective in either NER or MMR. Remarkably, cisplatin induced substantial levels of apoptotic cells in the *xpa-1(ok698*); *oct-1(RNAi*) (Figure S7G) and *msh-2(ok2410*)*; oct-1(RNAi*) (Figure S7I) mutants, when *oct-2* was upregulated (Figure S7L,M, respectively). These two DNA repair defective mutants were effectively protected from the onslaught of cisplatin-induced DNA lesions upon RNAi-driven depletion of *oct-2* (Figure S7G,I, respectively), consistent with the involvement of OCT-2 in the transport of cisplatin.

It is noteworthy that amongst the DNA repair deficient animals, only in the case of the *apn-1(tm6691*)*; oct-1(RNAi*) genotype was there a significant increase in spontaneous germ cell death (Figure S7D). One possible explanation for this observation is that OCT-2 mediated uptake of prooxidants may lead to oxidative lesions that must be repaired by BER^28^. Collectively, the above data suggest that by combining defects in DNA repair pathways with functional organic cation transporters such as OCT-2, it is possible to determine whether an unknown compound has genotoxic effects and the type of lesions it may create.

### Ligand-protein docking analysis predicts the substrate specificity of OCT-2

**Figure 4:**
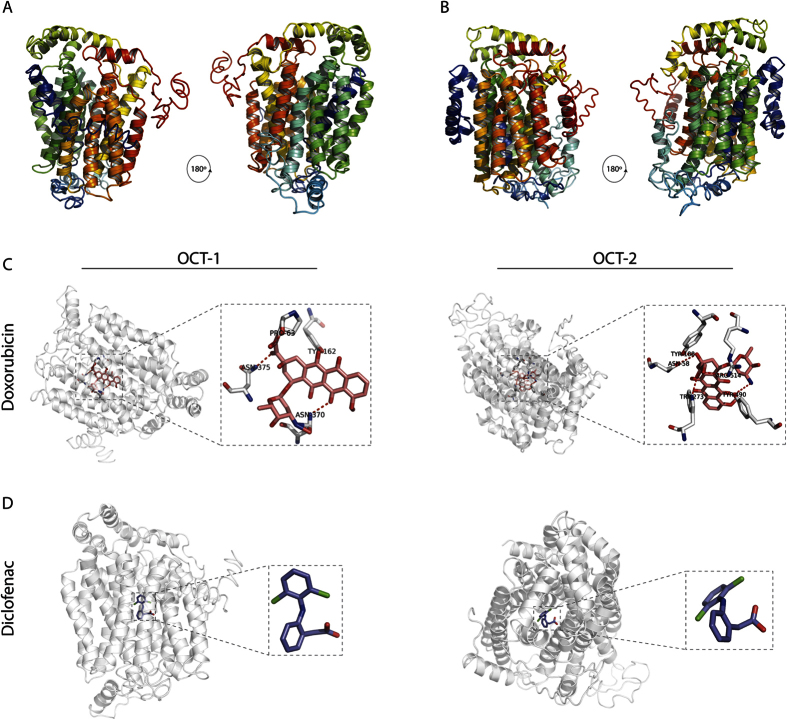
Structural modelling and protein-ligand docking of *C. elegans* OCT-1 and OCT-2. (**A,B**) Structural models of OCT-1 and OCT-2 were generated on the basis of GLUT3 (PDB ID: 5c65) structure and sequence conservation. N-terminal and C-terminal are coloured blue and red, respectively. (**C,D**) Predicted binding models of OCT-1 and OCT-2 with cationic and anionic ligands doxorubicin (pink) and diclofenac (purple), respectively. Residues making polar contacts with the ligands are depicted with sticks and represented with dotted red lines; oxygen atoms are coloured in red, nitrogen atoms in blue and carbon atoms in white. All representative figures were rendered with PyMol.

To gather insights into the substrates that can be recognized by OCT-2, we first made predictions of its protein structure relative to OCT-1 by inputting the respective primary protein sequences into the I-TASSER protein structure prediction server for *in silico* analyses^31^. The I-TASSER server employed known Protein Data Bank (PDB) structures as threading templates to predict the OCT-1 and OCT-2 structures (Table S1A,B). OCT-1 and OCT-2 were both modeled based on the X-ray diffraction structure of the glucose transporter GLUT3/SLC2A3 from *Homo sapiens* (PDB ID: 5c65) and validated with the glucose transporters GLUT1–4 structures (PDB ID: 4gc0) (Table S2A,B)^32^. The analysis revealed that OCT-1 and OCT-2 have predicted structures similar to each other (Fig. 4A,B). The comparative models of OCT-1 and OCT-2 were computed by utilizing several criteria as described in the materials and methods (Table S3). The final predicted 3D structure of OCT-1 and OCT-2 featured the entire 12 transmembrane domain helices (Figure S8A,B). Overall, the analysis predicted structures for both OCT-1 and OCT-2 that belong to solute carrier transporter family (Fig. 4A,B).

We next determined whether the anticancer drugs, doxorubicin and cisplatin, would dock onto the predicted structures of OCT-1 and OCT-2. We utilized the BSP-SLIM and COACH algorithms^33^,^34^ to predict the amino acid residues of the transporters constituting the ligand–protein docking sites (Fig. 4C and Table S4). Since the BSP-SLIM server did not recognize the cisplatin chemical structure, a related member of the platinum drug family, carboplatin, was used. The OCT-1 and OCT-2 models interacting with doxorubicin computed a C-score of 0.38 and 0.75, respectively (Fig. 4C) (see materials and methods, Table S4). The predicted key residues mediating interaction between the drugs and the transporters suggest that the binding pockets of OCT-1 and OCT-2 are structurally different (Fig. 4C), making the ligand-protein binding highly selective depending upon the chemical structure of the ligand.

The OCT-1 and OCT-2 models were also evaluated for their ability to discriminate between the interacting ligands and probable non-binder compounds. As non-binder compounds, we targeted the nonsteroidal anti-inflammatory drug diclofenac, which is classified as an organic anion. The predicted models showed that no residues of OCT-1 or OCT-2 interact with diclofenac (Fig. 4D and Table S5). Thus, it is possible to perform protein-ligand modeling studies in order to predict and uncover novel substrates for uptake by these transporters, which could be further corroborated by the above *in vivo* assays.

### Predicted ligands of OCT-1 and OCT-2 and experimental validation

So far, the roles of transporters in the uptake of a vast majority of genotoxic cationic drugs have not been tested in *C. elegans*. We sought to identify which of the known cationic drugs would be taken up by OCT-1 and/or OCT-2. A number of drugs were selected based on two criteria (i) mechanism of action and (ii) biological response. We assessed the docking ability of each compound with OCT-1 and OCT-2. From 19 tested ligands, four resulted with a docking score of zero, whereas the remaining 15 revealed a docking score > 3.5 favouring avid binding with OCT-2 (Table 1 dataset, docking score columns). Amongst these 15 ligands, some possess distinct pharmacological attributes such as creating different types of DNA lesions (Table S6). We next experimentally validated the *in silico* analyses using our drug-induced apoptosis assay as readout. We found that a number of the compounds that docked onto OCT-2 were capable of triggering high level of apoptotic cell death when *oct-2* was upregulated by *oct-1(RNAi*), as compared to the control RNAi (Table 1 dataset; numbers in the bracket are from control RNAi). There were also ligands that act by damaging the DNA such as melphalan and methoxyamine, which did not dock onto OCT-2, but induced apoptotic corpses (Table 1 dataset). We postulate that these non-binders might use alternative transporters to enter the animal.

**Figure 5:**
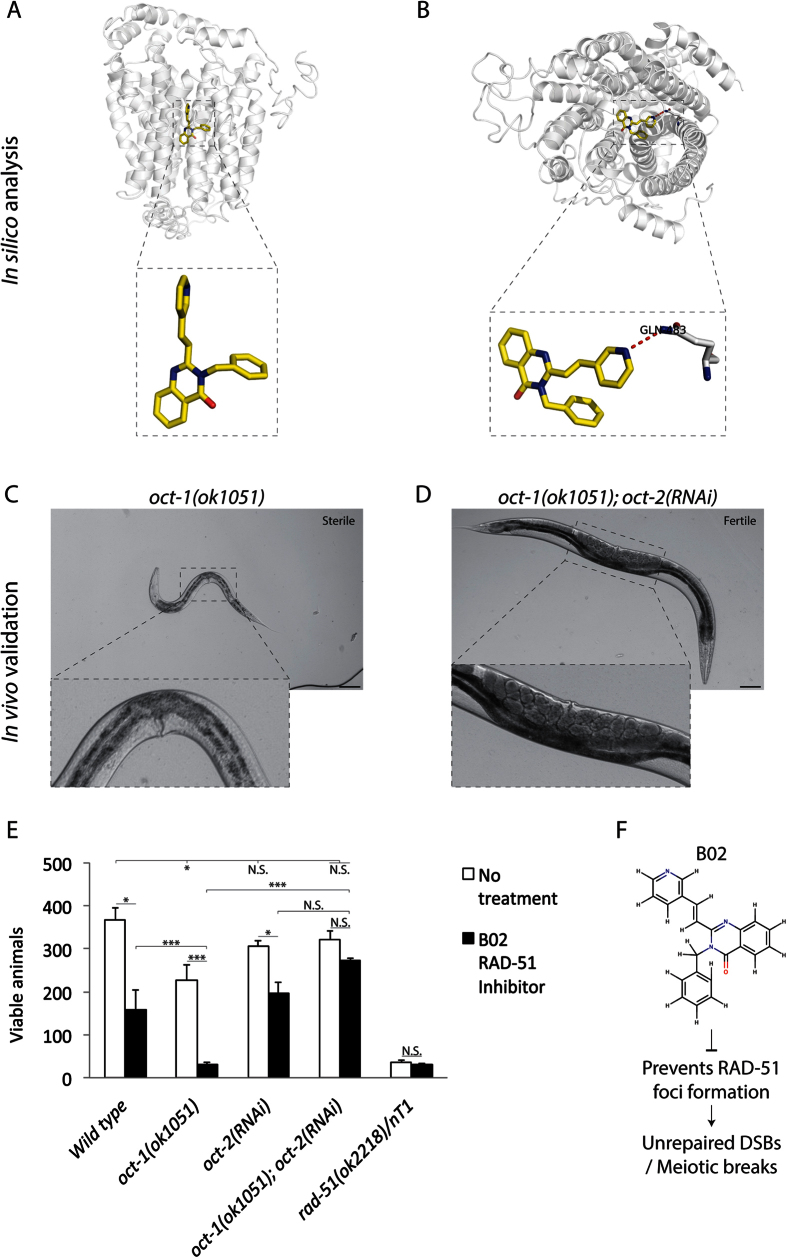
The B02 inhibitor of RAD-51 mimics the *rad-51/rad-51* homozygotes phenotypes when *oct-2* is upregulated. (**A,B**) Depiction of the ligand-protein docking of OCT-1 and OCT-2 modelled with B02 and showing exclusive binding of the drug to OCT-2. (**C**) DIC image showing that B02 causes embryonic lethality (diminishing number of germ cells) in the *oct-1(ok1051*) mutant. (**D**) DIC image showing that *oct-2* downregulation in the *oct-1(ok1051*) mutant animals restores fertility. In (**C,D**) Scale bar = 35 μm. (**E**) B02 diminishes the number of viable animals when *oct-2* is expressed. Brood size analysis of wild type, *oct-1(ok1051*) and *oct-1(ok1051*)*; oct-2(RNAi*) and *rad-51(ok2218*)*/nT1* animals under standard conditions (no treatment) and upon exposure to 5 μM B02. Data are mean ± S.D. No treatment: Wild type = 366 ± 26.7 (*n* = 20), *oct-1(ok1051*) = 296 ± 7.1 (*n* = 29), *oct-2(RNAi*) = 307 ± 11.3 (*n* = 23), *oct-1(ok1051*)*; oct-2(RNAi*) = 321 ± 19.7 (*n* = 25), *rad-51(ok2218*)*/nT1* = 35 ± 5.6 (*n* = 9). B02 treatment (5 μM): Wild type = 158 ± 29.7 (*n* = 21), *oct-1(ok1051*) = 32 ± 7.0 (*n* = 17), *oct-2(RNAi*) = 196 ± 26.8 (*n* = 25), *oct-1(ok1051*)*; oct-2(RNAi*) = 272 ± 7.1 (*n* = 25), *rad-51(ok2218*)/*nT1* = 30 ± 4.2 (*n* = 10). Error bars represent the S.D. Unpaired two-tail t-test *P < 0.03; **P < 0.01; ***P < 0.0005 were considered to be statistically significant. N.S. = Non Significant. (**F**) The RAD-51 inhibitor, B02, mode of action in *C. elegans* germline.

As a final validation, we focused on the ligand B02, which robustly docked only onto OCT-2 with a docking score of 5.4 (Table 1 dataset, Fig. 5A vs. B). B02 was shown to interfere with human RAD51 in DNA strand exchange and nuclear focus formation in response to DNA damage^35^,^36^,^37^. However, to our knowledge the pharmacological effect of B02 has not been tested in *C. elegans*. To confirm that B02 enters via the OCT-2 transporter, we tested the effect of the compound on wild type, *oct-1(ok1051*) and the *oct-1(ok1051*)*; oct-2(RNAi*) backgrounds at concentrations ranging from 1 to 75 μM. Notably, extreme toxicity was observed with 75 μM B02 for all the genotypes. Therefore, we reduced the concentrations to 5 μM and observed that *oct-2* upregulation caused sterility (Fig. 5C) resulting in a decrease in viable animals (Fig. 5E) and phenocopying the *rad-51* homozygotes as previously reported^38^. As predicted from our model, the B02 ligand did not cause reduction in viable animals in the *oct-1(ok1051*)*; oct-2(RNAi*) genetic background (Fig. 5D) and restored the number of broods to nearly untreated levels (Fig. 5E). These compelling data revealed for the first time that the B02 inhibitor of RAD51 is functionally active in *C. elegans* and requires exclusive uptake by OCT-2. We conclude that the docking scores of ligands and quantifiable endpoints provide valuable tools to monitor transporter-mediated drug uptake into *C. elegans.* This approach is particularly suitable for newly developed drugs that cannot be readily labeled or lack fluorescent properties for uptake studies.

## Discussion

In this study, we established for the first time the function of OCT-2 in *C. elegans*, as well as the specific roles for OCT-1 and OCT-2 in mediating tissue transport of distinct compounds, such as the chemotherapeutic drugs anthracyclines and cisplatin. We show that OCT-1 has no direct role in the transport of these compounds, however, it exerts control on *oct-2* expression, and it is OCT-2 that is primarily involved in the uptake of these agents. This conclusion is derived from three key findings. First, the *oct-1* deletion mutant *oct-1(ok1051*) or RNAi-driven knockdown of *oct-1* triggered the upregulation of *oct-2* in the whole body, the head and gonads of the animal, causing hypersensitivity to chemotherapeutic drugs. This phenotype is observed only when OCT-2 is present. Second, RNAi-driven knockdown of *oct-2* blocked uptake of chemotherapeutic drugs thus preventing their genotoxic effects. Since no additional drug resistance was observed in *oct-1(ok1051*); *oct-2(RNAi*) double mutant animals as compared to ones depleted for *oct-2* alone, a role for OCT-1 in the uptake of these chemotherapeutic drugs can be excluded; nonetheless OCT-1 could act as a transporter for selective ligands. And third, our *in silico* modeling-based screening of OCT-1 and OCT-2 selectively discriminated amongst DNA damaging agents those that are potential ligands for OCT-2. By validating this approach *in vivo*, we show that OCT2-dependent transport of the DNA damaging agents can sensitize mutant animals that are defective in DNA repair pathways. Collectively, these results underscore the importance of uptake transporters in regulating the entry of chemotherapeutic drugs into cells and raise the possibility that the drug-resistance and drug-sensitive responses observed by cancer patients could be governed at the level of drug uptake.

The downregulation of *oct-1* leading to upregulation of *oct-2* was an unexpected finding, and provides a compelling argument that the animal has evolved tight regulation of OCT-2. So how might OCT-1 depletion lead to the activation of OCT-2? One possibility is that both transporters have the ability to transport common essential nutrients and thus deletion of *oct-1* would stimulate OCT-2 to compensate for the deprivation of pivotal nutrients. However, we argue against this possibility, as depletion of both *oct-1* and *oct-2* expression did not result in animals with any dramatic phenotypes under normal growth conditions. A more likely possibility is that OCT-1 might belong to the recent characterized class of surface sensors that act as non-transporting transceptors by sensing the availability of nutrients and signal the regulation of downstream plasma membrane transporters^39^,^40^. In this model, when nutrients become scarce, OCT-1 might serve as a sensor to promote the upregulation of OCT-2 to scavenge limiting resources. Conversely, when nutrients are plentiful OCT-1 might function to sustain the basal expression of OCT-2. Precedence for this mode of regulation exists in *S. cerevisiae*, *Drosophila melanogaster* and *Homo sapiens*^41^. For instance, in *S. cerevisiae* the Ssy1 sensor, a plasma membrane protein belonging to the amino acid permease family, is endowed with no, or only limited, transport function^42^. Ssy1 senses amino acid availability by direct interaction with extracellular amino acids and transmitting the signal to zinc-finger transcription factors that trigger the expression of several downstream target genes encoding amino acid permeases^42^,^43^,^44^. Similar sensors exist in mammalian cells, e.g., the SGLT3 glucose sensor that binds, but does not transport, sugar molecules^45^. Thus, in view of the increasing number of sensors that are currently being identified, it is plausible that OCT-1 may indeed act either as a non-transporting or transporting sensor leading to regulation of OCT-2 expression. The exact role by which OCT-1 exerts control on OCT-2 will need further investigation, but it is noteworthy that a similar regulation appears to occur in mice where deletion of *OCT1* causes significant upregulation of mRNA transcripts of its homologues *OCT2* and *OCT3*^9^.

Remarkably, *oct-2* downregulation rescued shortened lifespan and sharply decreased spontaneous apoptosis observed in the *oct-1(ok1051*) deletion mutant (Figs 1B and 3B). The most direct interpretation is that OCT-2 indiscriminately transports toxic compounds such as prooxidants. Although the source of these compound(s) is unknown, i.e., whether they originate from the feeding bacteria or re-adsorption of metabolites secreted by *C. elegans*, they are capable of inducing DNA lesions that must be removed by BER. This is supported by the observation that only the BER defective *apn-1* deletion mutant exhibited higher levels of spontaneous apoptosis when *oct-2* expression is upregulated. Thus, the previous report showing that *oct-1* deletion animal exhibit a decline in lifespan due to reduced uptake of the antioxidant ergothioneine can alternatively be explained if the sulphur atom on the imidazole ring of ergothioneine serves to detoxify the OCT-2-dependent uptake of prooxidants^2^. Nonetheless, our findings raise a very important concern regarding genetic variations leading to hyperactivation of uptake transporters as previously reported^46^. This hyperactivation likely to cause accumulation of abnormally high concentrations of genotoxic compounds and metabolites. Consequently, such toxic agents could induce substantial DNA damage over the lifetime of an individual causing genomic instability and eventually cancer.

Our study is the first to demonstrate that *C. elegans* OCT-2 plays a role in ligand uptake. We tested doxorubicin and cisplatin as the initial cationic ligands because they are first-line chemotherapeutics believed to be transported by OCTs in human cells^4^,^47^. Besides these anticancer drugs, we postulate that OCT-2 may recognize a vast array of other cationic compounds. *Per se*, we implemented the OCT-based ligand-protein docking approach and explore a short list of selected cationic compounds to deduce that OCT-2 was promiscuous compared to OCT-1 and that it interacts robustly with several cationic ligands. Importantly, the analysis produced a refined list of genotoxic compounds that display high protein-ligand docking scores all of which show an OCT-2 dependent *in vivo* effect of triggering germ cells apoptosis. We believe that exploiting OCT-2 in *C. elegans* could have far reaching applications and supersede other whole model systems in drug discovery programs with respect to cost and time. Thus, maintaining the OCT-2 transporter at optimal levels by deleting *oct-1* should represent a useful step for incorporation into any high-throughput screens to more efficiently identify bioactive molecules from chemical libraries. A key aspect of this strategy is that overexpressed OCT-2 is expected to operate with significantly lower chemical concentrations as observed with cisplatin where a fixed lower concentration of the drug had no effect on the wild type, but significantly induced apoptosis in the *oct-1(ok1051*) mutant (Figure S9). Thus, the previous barriers posed by *C. elegans* to find bioactive molecules could be explained by the lack of an activated mechanism to efficiently take up the compounds at lower concentrations. In short, we now provide a comprehensive readout of the OCT-2 functional selectivity towards cationic molecules that have a deleterious effect on *C. elegans,* and therefore provide a foundation to understand the regulatory control of drug uptake to circumvent genotoxicities. In addition, we hypothesize that OCT-2 could be exploited either through the *oct-1* gene deletion mutant or *oct-2* over-expression transgenic animals to generate a hypersensitive ‘screening’ *C. elegans* to facilitate high-throughput drug screening.

## Materials and Methods

### Nematode strains and culture conditions

The Bristol N2 (wild type), RB1084 [*oct-1(ok1051*) I], VC1973 [*rad-51(ok2218*) IV/nT1 [qIs51] (IV;V)]*, RB864 [*xpa-1(ok698*) I], RB1864 [*msh-2(ok2410*) I], MD701 [*bcIs39* [lim-7p::ced-1::GFP + lin-15(+)] and DA453 [*eat-2(ad453*) II], TJ1 [*cep-1(gk138*) I], MT1082 [*egl-1(n487*) V], MT4770 [*ced-9(n1950*) III], MT5287 [*ced-4(n1894*) III], MT3002 [*ced-3(n1286*) IV] and CL2166 [*dvIs19* [(pAF15) gst-4p::GFP::NLS] III] *Caenorhabditis elegans* strains were obtained from the CGC Stock center (*Caenorhabditis* Genetics Centre, University of Minnesota, Minneapolis, USA). The [*apn-1(tm6691*) II] were obtained from Shohei Mitani (Tokyo Women’s Medical University School of Medicine, Japan and the National Bioresource Project for the nematode *C. elegans*). The alleles used in this study were all previously validated to be null. All *C. elegans* strains were maintained at 20 °C on nematode growth medium (NGM) agar (2.5 g/L peptone, 51.3 mM NaCl, 17 g/L agar, 1 mM CaCl_2_, 1 mM MgSO_4_, 25 mM KPO_4_, and 12.9 μM cholesterol) enriched with a lawn of streptomycin-resistant *Escherichia coli* OP50 bacterial strain as a source of food. For all *in vivo* experiments, developmental staged-synchronized nematodes were obtained by hypochlorite treatment of gravid adult hermaphrodites. Eggs were allowed to hatch on M9 buffer (6g Na_2_HPO_4_, 3g KH_2_PO_4_, 5g NaCl, 0.25g MgSO_4 _· 7H_2_O per liter filter sterilized). In all experiments, animals were monitored from day 1 post-L1 larvae stage and from L4 to avoid experimental bias. *Homozygous *rad-51/rad-51* nematodes^27^ show almost complete inviability due to high embryonic lethality in their progeny, thus we analyzed heterozygote nematodes due to the ease of RNAi-feeding for further analyses. *C. elegans* strains were backcrossed at least three times.

### Lifespan assay

Lifespan assays were performed at 20 °C in standard conditions and assessed blindly as previously described^10^.

### Drug treatment

The anthracycline doxorubicin, water-soluble platinum complex cisplatin and alkylating agent methyl methanesulfonate (Sigma Cat. N° 129925) were added to the NGM agar medium before solidification (~55 °C) to obtain a final concentration of 100 μM for doxorubicin and cisplatin and 0.25 μM for methyl methanesulfonate, respectively. For all experiments, L1-staged from F1 synchronized nematodes were transferred to NGM control agar plates and containing doxorubicin, cisplatin and methyl methanesulfonate. Doxorubicin and cisplatin working concentrations were chosen based on previously reported assays^14^. All drug-containing plates were freshly made prior to each experiment. Our oncology pharmacy department (Maisonneuve-Rosemont Hospital (HMR) provided doxorubicin and cisplatin.

### Microscopy and imaging

All microscopy was performed utilizing a DeltaVision Elite Image Restoration System (Applied Precision) with either 40x/0.65–1.35 or 63x/1.42 oil objective. The worms were anesthetized with levamisole (5 μM, Sigma Cat. N° L0380000) and mounted on 2% agarose pads for their respective imaging and quantification. Images were processed utilizing ImageJ imaging software^48^.

### DNA damage response assay and germ cells imaging

The methods previously described were used^21^. Briefly, to quantify the number of apoptotic corpses in nematodes, L1-staged synchronized N2 wild type, *oct-1(ok1051*) and DNA repair deficient mutants were exposed to different doses of drugs followed by germ cells apoptosis assay. Between 18 to 24 hours past L4-staged nematodes, adult moult staged nematodes were assayed with differential interference contrast (DIC) microscopy (Nomarski) optics and the vital DNA dye acridine orange (Sigma Cat. N° A6024). Nematodes were incubated in the dark for 2 hours at 20 °C on NGM plates containing 1 ml of 50 μg/μl of acridine orange DNA dye dissolved in M9 buffer. Stained nematodes were transferred to fresh OP50-seeded NGM plates to incubate for 2 hours in order to clear off the stained bacteria. The acridine orange-stained and DIC-visible apoptotic corpses were counted with an exposure time of 1 second and 0.8 seconds, respectively. The engulfment of apoptotic corpses was scored utilizing the CED-1::GFP reporter and imaged similarly with an exposure time of 1 second utilizing the GFP channel. Images were collected as a series of 25/0.5 μm optical sections covering the complete thickness of the gonad arm.

### Imaging the oxidative stress-inducible GFP reporter GST-4::GFP

The uptake of prooxidants was detected by imaging the oxidative stress-inducible GFP reporter GST-4::GFP. L4-staged synchronized nematodes were maintained in NGM plates at 20 °C overnight and imaged the next day at the adult moult stage. The image analysis was performed by measuring the GFP fluorescence intensity of the whole animal utilizing the GFP channel exposed for 0.8 seconds and DIC exposed for 0.05 seconds.

### Imaging the pharynx to measure the uptake of doxorubicin

To measure the uptake of doxorubicin, an approach focusing on the pharynx of *C. elegans* was developed. L4-staged synchronized nematodes were treated with doxorubicin 100 μM in NGM plates, incubated at 20 °C overnight and imaged the next day at the adult moult stage. The image analysis was performed by measuring the doxorubicin fluorescence intensity (λ^ex^470 nm–λ^em^585 nm) localized at the pharynx by utilizing the GFP channel and observed under a 40x/0.65–1.35 oil objective exposed for a period of time of 0.5 seconds. ImageJ^48^ imaging software was utilized to determine the level of fluorescence in the pharynx region. The data of measured fluorescence intensity for doxorubicin uptake was depicted by implementing the custom built-in interactive 3D Surface Plot featured in ImageJ, which display the intensities of pixels from a region of interest of a given image. The uptake was corroborated by two means: (i) The area, the integrated density and the mean gray value were considered to calculate the corrected total pharynx fluorescence [CTPF = Integrated Density − (Area of selected pharynx X Mean fluorescence of background readings)]^49^, and (ii) Fluoroskan analysis (see below). To measure doxorubicin uptake in a dose-dependent manner, the nematodes were treated with 1 ml of varying concentrations (10 to 100 μM) of the drug in M9 buffer. Uptake of Fluorescein (Sigma Cat. N° F2456), at concentrations ranging from 1 to 100 μM, was used as a control.

### Relative RNA quantification to monitor gene expression

Total RNA (RNeasy mini kit Qiagen Cat. N° 74104) was prepared from ~1000 L4 synchronized nematodes and used for cDNA synthesis (Invitrogen Cat. N° 28025-013) followed by quantitative real-time PCR (qRT-PCR). qRT-PCR was performed with the Supergreen Mastermix (Wisent Bioproducts Cat. N° 800-431-UL) starting at 95 °C for 2 min, followed by 40 cycles at 95 °C for 5 sec, 63 °C for 30 sec and 72 °C for 30 sec. Transcript levels were normalized to the internal controls *act-1* and *pmp-3* encoding actin and the peroxisomal membrane protein, respectively. Because there is a putative third organic cationic transporter PES-23, which shares 21.31% and 17.18% identity with OCT-1 and OCT-2, respectively, we monitored *pes-23* gene expression for off targets. The forward and reverse primer sequences utilized in this study were: *oct-1*: 5′-TTTGGAGCAGCTATGGCTTT-3′ and 5′-CTTAGCGTCAGCCCATTTTC-3′; *oct-2*: 5′-TTGGAGTCGTGCTCACGTTC-3′ and 5′-GAGTATGTGAGAAGAAAGCC-3′; *act-1*: 5′-TGCTGATCGTATGCAGAAGG-3′ and 5′-TAGATCCTCCGATCCAGACG-3′; *pmp-3*: 5′-GTTCCCGTGTTCATCACTCAT-3′ and 5′-ACACCGTCGAGAAGCTGTAGA-3′; *pes-23*: 5′-TTCTTGCCGGAGTACCTGCC-3′ and 5′-GCACACATGGAGATTCCGTT-3′.

### Dissection of C. elegans heads and gonads for relative RNA quantification

Nematodes were first transferred to a dried 2% agarose pad in 10 μl of M9 buffer. Exactly 300 N2 wild type and 300 *oct-1(ok1051*) L4 ~ young adult moult staged nematodes were decapitated just posterior to the pharynx by utilizing a 26^1/2^ gauge syringe. Severed heads were washed and collected with M9 buffer in a micro-centrifuge tube and rapidly stored at −80 °C in Trizol (Ambion Life Technologies Cat. N° 15596-018). The same procedure was followed to dissect 100 gonads from L4 ~ young adult staged nematodes. In order to preserve optimum tissue integrity, the collection was made every 15 severed heads and every 10 gonads respectively. RNA extraction was performed from the pool of all collected severed heads and dissected gonads and stored at −80 °C for further qRT-PCR analysis.

### RNA interference analysis

*Escherichia coli* HT115^DE3^ strain harboring specific RNAi constructs against *oct-1* and *oct-2* was grown on lysogeny broth (LB) agar plates containing ampicillin and tetracycline. Overnight cultures were grown in LB media containing ampicillin. For *oct-1* and *oct-2* RNAi-driven knockdown experiments, nematodes were maintained until first generation (F1) on NGM agar plates containing 1 mM IPTG (isopropyl-β-D-1-thiogalactopyranoside) enriched with a lawn of *E. coli* HT115^DE3^ expressing RNAi constructs in the pL4440-feeding vector at standard temperature 20 °C. For *oct-1* and *oct-2* RNAi-driven knockdown efficiency, mRNA expression levels were measured in synchronized L4-staged collected from the F1 generation of nematodes fed with *E. coli* expressing RNAi targeted to the indicated genes. The RNAi clones were obtained from the Ahringer laboratory library^50^ and verified by sequencing. The depletion efficiency of *oct-1* and *oct-2* genes was validated by qRT-PCR. In all experiments synchronized L4 animals were fed RNAi expressing bacteria and the resulting F1 animals were analyzed for phenotypes.

### Choline-based competition assay

L4-staged F1 nematodes were treated overnight with 50 μM and 100 μM concentrations of choline and doxorubicin as separate conditions and together in equimolar amounts in NGM agar plates. The differences in wavelength indexes between doxorubicin (λ^ex^470 nm–λ^em^585 nm) and choline (λ^ex^290 nm–λ^em^345 nm), allowed us to measure the competitive uptake in the pharynx utilizing the GFP wavelength index where only doxorubicin is detectable and not choline.

### Statistical analyses

Lifespan analyses were performed utilizing the Kaplan-Meier estimator calculating the Log-rank test for statistical significance utilizing OASIS software (Online Application for the Survival Analysis of Lifespan Assays Performed in Aging Research)^51^. Germ cell death statistical significance was assessed with the Mann-Whitney U-test calculator Mean values ± s.e.m were calculated for each condition. *P < 0.05; **P < 0.01; ***P < 0.001; ****P < 0.0001 were considered to be statistically significant. For the Brood size analysis, statistical differences were calculated by the unpaired two-tail t-test (*P < 0.03; **P < 0.01; ***P < 0.0005) and represented as ± S.D. The Fluoroskan data and the Mean Fluorescence Intensity measurements extracted from the competition assay, Student T-test was calculated and represented as ± S.D (***P < 0.001 significant). N.S. = Non Significant. Statistical differences were calculated by using the GraphPad Prism Statistical Software Mac Version 6.

### Comparative structure and model construction

*C. elegans* OCT-1 (F52F12.1) and OCT-2 (ZK455.8) putative protein sequences were first obtained from the Wormbase. OCT-1 and OCT-2 were modeled using I-TASSER (Iterative Threading ASSEmbly Refinement)^31^ which utilized the best 10 threading template structures from distinct species from the PDB database (Table S1a,b). We relied on the top 5 threading templates ranked by their identity and Z-score, where a Z-score higher than 1 signified a correct alignment. The final models of OCT-1 and OCT-2 were assessed based on the X-ray diffraction structural analogs of the glucose transporter GLUT3 (PDB ID: 5c65) from *Homo sapiens* (Table S2a,b). The models took into account the following (i) the C-score criteria, a confidence score for estimating the quality of predicted models and TM-score criteria, a metric measurement of the structural similarity between two protein models from I-TASSER^52^, (ii) the TM-score from ModRefiner^53^ and (iii) the Z-DOPE from Modeller, an atomic distance-dependent statistical calculation from samples of native protein structures that does not depend on any adjustable criteria^54^. These final predicted structures were also assessed based on primary sequence alignment and evolutionary conservation profiles using the PROMALS3D multiple sequence and structure alignment server^55^. Finally, for the predictions of the transmembrane domains, the ResQ *B*-factor profile provided a consensus prediction where the secondary structure helices (SS) are depicted as red tubes^56^ (Figure S8A,B), and corroborated with the Orientation of Protein in Membranes (OPM) server^57^, the Dense Alignment Surface (DAS) method^58^ and the Open-source tool for visualization of proteoforms (PROTTER)^59^.

### Ligand-protein docking

The ligand chemical structures for doxorubicin (ID: 31703) and diclofenac (ID: 3033) were obtained from the PubChem database^60^. Ligand-protein docking were performed through the BSP-SLIM (Binding Site Prediction with Shape-based Ligand Matching with binding pocket) and COACH algorithms^33^,^34^, featured in the I-TASSER unified platform, to predict the residues constituting the ligand–protein docking sites and conformations of OCT-1 and OCT-2 with doxorubicin and diclofenac.

### Structure visualization

OCT-1 and OCT-2 three-dimensional structures and protein-ligand interacting structures were visualized using the OpenGL PyMOL Molecular Graphics System, Mac Version 1.7.4 Schrödinger, LLC.

### *In vivo* validation of the predicted ligand-protein docking models

The ligand-protein docking was performed utilizing the BSP-SLIM server and validated following the same strategy as described in the drug treatment and DNA damage response assay and germ cells imaging sections. Synchronized L1-staged worms were exposed to the following compounds dissolved in DMSO; the RAD-51 inhibitor B02 (5 μM) (ID: 5738263) (EMD Millipore Cat. N° 553525), Camptothecin (75 μM) (ID: 24360) (Sigma Cat. N° C9911), Cycloheximide (50 μM) (ID: 6197) (Sigma Cat. N° C7698), Ketamine (ID: 3821) (predicted virtually), Melphalan (ID: 460612) (predicted virtually), and these additional compounds dissolved in water; Metformin (75 μM) (ID: 4091), Methotrexate (50 μM) (ID: 126941), Methoxyamine (10 μM) (ID: 4113) (Santa Cruz Biotechnology Cat. N° SC257710), Methyl methane sulfonate (0.25 μM) (ID: 5156) (Sigma Cat, N° 129925), Nicotinamide (100 μM) (ID: 936) (Sigma Cat. N° N3376), 4-Nitroquinoline N-oxide (75 μM) (ID: 5955) (ICN Biomedicals Cat. N° 15596), Olaparib (ID: 23725625) (predicted virtually), Paraquat (100 μM) (ID: 15939) (Sigma Cat. N° 856177), Phenformin (75 μM) (Sigma Cat. N° P7045), Puromycin (100 μM) (ID: 439530) (Sigma Cat. N° P9620), Zeocin (25 μM) (ID: 71668282) (Santa Cruz Biotechnology Cat. N° SC496345), and analyzed at the young adult stage. Methotrexate and Metformin were obtained from our oncology pharmacy department (Maisonneuve-Rosemont Hospital (HMR). A concentration of 0.2% DMSO was used for control plates. All drug-treated plates were made fresh and seeded with bacteria 12 hours prior to each experiment.

### Brood size quantification to validate the effects of the RAD-51 inhibitor, B02

Single L1-staged worm from wild type and mutant genotypes were transferred to seeded NGM plates without and with B02 and maintained at 20 °C. Animals were transferred to fresh plates each day until they stopped laying eggs. The hatched larvae on each plate were counted and total number of viable larvae that developed to the L1 stage descended from a single hermaphrodite was counted. The average number of viable larvae from 10 to 25 animals of a strain was plotted as brood size where the progeny is allowed to reach adulthood and scored as being fertile or sterile. The brood size of doxorubicin-treated animals was performed similarly where unhatched and hatched progeny was plotted as the total brood size. Statistical differences were calculated by unpaired two-tail t-test (*P < 0.03; **P < 0.01; ***P < 0.0005; N.S. = Non Significant) and represented as ± S.D using GraphPad Prism Statistical Software.

### Fluoroskan analysis

Synchronized L1-staged nematodes were exposed to doxorubicin (100 μM) as described above. L4 ~ young adult-staged nematodes were washed at least two times with M9 buffer prior to quantification. A total of 50 worms were placed into each well in duplicate in a 96 format black-well plate with optical bottom (Fisher Scientific). Doxorubicin uptake was measured with a microplate fluorometer (Fluoroskan Ascent, Thermo Scientific, USA) utilizing λ^ex^544 nm–λ^em^590 nm filters. Fluorescein 1 μM was used as an internal control for all genotypes. Student T-test was calculated and represented as ± S.D ***P < 0.001 statistical significance and N.S. = Non Significant.

## Additional Information

**How to cite this article**: Papaluca, A. and Ramotar, D. A novel approach using *C. elegans* DNA damage-induced apoptosis to characterize the dynamics of uptake transporters for therapeutic drug discoveries. *Sci. Rep.* **6**, 36026; doi: 10.1038/srep36026 (2016).

**Publisher’s note:** Springer Nature remains neutral with regard to jurisdictional claims in published maps and institutional affiliations.

## Supplementary Material

Supplementary Table 1

Supplementary Information

## References

[b1] O’Reilly L. P., Luke C. J., Perlmutter D. H., Silverman G. A. & Pak S. C. C. elegans in high-throughput drug discovery. Adv Drug Deliv Rev 69–70, 247–253 (2014). 10.1016/j.addr.2013.12.001 PMC4019719 24333896

[b2] Cheah I. K. *et al.* Knockout of a putative ergothioneine transporter in Caenorhabditis elegans decreases lifespan and increases susceptibility to oxidative damage. Free Radic Res 47, 1036–1045 (2013). 24074059 10.3109/10715762.2013.848354

[b3] Koepsell H. The SLC22 family with transporters of organic cations, anions and zwitterions. Mol Aspects Med 34, 413–435 (2013). 23506881 10.1016/j.mam.2012.10.010

[b4] Andreev E., Brosseau N., Carmona E., Mes-Masson A. M. & Ramotar D. The human organic cation transporter OCT1 mediates high affinity uptake of the anticancer drug daunorubicin. Sci Rep 6, 20508 (2016). 26861753 10.1038/srep20508 PMC4748219

[b5] Wu X. *et al.* Identity of the F52F12.1 gene product in Caenorhabditis elegans as an organic cation transporter. Biochim Biophys Acta 1418, 239–244 (1999). 10209228 10.1016/s0005-2736(99)00020-6

[b6] Brosseau N., Andreev E. & Ramotar D. Complementation of the Yeast Model System Reveals that Caenorhabditis elegans OCT-1 Is a Functional Transporter of Anthracyclines. PLoS One 10, e0133182 (2015). 26177450 10.1371/journal.pone.0133182 PMC4503637

[b7] Kaletta T. & Hengartner M. O. Finding function in novel targets: C. elegans as a model organism. Nat Rev Drug Discov 5, 387–398 (2006). 16672925 10.1038/nrd2031

[b8] Burns A. R. *et al.* A predictive model for drug bioaccumulation and bioactivity in Caenorhabditis elegans. Nat Chem Biol 6, 549–557 (2010). 20512140 10.1038/nchembio.380

[b9] Chen L. *et al.* OCT1 is a high-capacity thiamine transporter that regulates hepatic steatosis and is a target of metformin. Proc Natl Acad Sci USA 111, 9983–9988 (2014). 24961373 10.1073/pnas.1314939111 PMC4103324

[b10] Lionaki E. & Tavernarakis N. Assessing aging and senescent decline in Caenorhabditis elegans: cohort survival analysis. Methods Mol Biol 965, 473–484 (2013). 23296678 10.1007/978-1-62703-239-1_31

[b11] McKay S. J. *et al.* Gene expression profiling of cells, tissues, and developmental stages of the nematode C. elegans. Cold Spring Harb Symp Quant Biol 68, 159–169 (2003). 15338614 10.1101/sqb.2003.68.159

[b12] Fishel M. L. *et al.* Apurinic/apyrimidinic endonuclease/redox factor-1 (APE1/Ref-1) redox function negatively regulates NRF2. J Biol Chem 290, 3057–3068 (2015). 25492865 10.1074/jbc.M114.621995 PMC4317024

[b13] Cabreiro F. *et al.* Metformin retards aging in C. elegans by altering microbial folate and methionine metabolism. Cell 153, 228–239 (2013). 23540700 10.1016/j.cell.2013.02.035 PMC3898468

[b14] Sendoel A. *et al.* DEPDC1/LET-99 participates in an evolutionarily conserved pathway for anti-tubulin drug-induced apoptosis. Nat Cell Biol 16, 812–820 (2014). 25064737 10.1038/ncb3010

[b15] Hall D. & Altun Z. C. elegans Atlas, (Cold Spring Harbor Laboratory Press, 2008).

[b16] McKay J. P., Raizen D. M., Gottschalk A., Schafer W. R. & Avery L. eat-2 and eat-18 are required for nicotinic neurotransmission in the Caenorhabditis elegans pharynx. Genetics 166, 161–169 (2004). 15020415 10.1534/genetics.166.1.161 PMC1470703

[b17] Hoffmann K., Grafe F., Wohlrab W., Neubert R. H. & Brandsch M. Functional characterization of a high-affinity choline transport system in human keratinocytes. J Invest Dermatol 119, 118–121 (2002). 12164933 10.1046/j.1523-1747.2002.01801.x

[b18] Gartner A., Boag P. R. & Blackwell T. K. Germline survival and apoptosis. WormBook, 1–20 (2008). 10.1895/wormbook.1.145.1 PMC4781258 18781708

[b19] Lans H. & Vermeulen W. Tissue specific response to DNA damage: C. elegans as role model. DNA Repair (Amst) 32, 141–148 (2015). 25957488 10.1016/j.dnarep.2015.04.025

[b20] Horvitz H. R. Genetic control of programmed cell death in the nematode Caenorhabditis elegans. Cancer Res 59, 1701s–1706s (1999). 10197583

[b21] Craig A. L., Moser S. C., Bailly A. P. & Gartner A. Methods for studying the DNA damage response in the Caenorhabdatis elegans germ line. Methods Cell Biol 107, 321–352 (2012). 22226529 10.1016/B978-0-12-394620-1.00011-4

[b22] Gumienny T. L., Lambie E., Hartwieg E., Horvitz H. R. & Hengartner M. O. Genetic control of programmed cell death in the Caenorhabditis elegans hermaphrodite germline. Development 126, 1011–1022 (1999). 9927601 10.1242/dev.126.5.1011

[b23] Savill J. & Fadok V. Corpse clearance defines the meaning of cell death. Nature 407, 784–788 (2000). 11048729 10.1038/35037722

[b24] Zhou Z., Hartwieg E. & Horvitz H. R. CED-1 is a transmembrane receptor that mediates cell corpse engulfment in C. elegans. Cell 104, 43–56 (2001). 11163239 10.1016/s0092-8674(01)00190-8

[b25] Li Z., Lu N., He X. & Zhou Z. Monitoring the clearance of apoptotic and necrotic cells in the nematode Caenorhabditis elegans. Methods Mol Biol 1004, 183–202 (2013). 23733578 10.1007/978-1-62703-383-1_14 PMC4038443

[b26] Lin L., Yee S. W., Kim R. B. & Giacomini K. M. SLC transporters as therapeutic targets: emerging opportunities. Nat Rev Drug Discov 14, 543–560 (2015). 26111766 10.1038/nrd4626 PMC4698371

[b27] Rinaldo C., Bazzicalupo P., Ederle S., Hilliard M. & La Volpe A. Roles for Caenorhabditis elegans rad-51 in meiosis and in resistance to ionizing radiation during development. Genetics 160, 471–479 (2002). 11861554 10.1093/genetics/160.2.471 PMC1461995

[b28] Yang X. *et al.* Functional characterization of the Caenorhabditis elegans DNA repair enzyme APN-1. DNA Repair (Amst) 11, 811–822 (2012). 22819077 10.1016/j.dnarep.2012.06.009

[b29] Minotti G., Menna P., Salvatorelli E., Cairo G. & Gianni L. Anthracyclines: molecular advances and pharmacologic developments in antitumor activity and cardiotoxicity. Pharmacol Rev 56, 185–229 (2004). 15169927 10.1124/pr.56.2.6

[b30] Wang D. & Lippard S. J. Cellular processing of platinum anticancer drugs. Nat Rev Drug Discov 4, 307–320 (2005). 15789122 10.1038/nrd1691

[b31] Roy A., Kucukural A. & Zhang Y. I-TASSER: a unified platform for automated protein structure and function prediction. Nat Protoc 5, 725–738 (2010). 20360767 10.1038/nprot.2010.5 PMC2849174

[b32] Sun L. *et al.* Crystal structure of a bacterial homologue of glucose transporters GLUT1-4. Nature 490, 361–366 (2012). 23075985 10.1038/nature11524

[b33] Lee H. S. & Zhang Y. BSP-SLIM: a blind low-resolution ligand-protein docking approach using predicted protein structures. Proteins 80, 93–110 (2012). 21971880 10.1002/prot.23165 PMC3240723

[b34] Yang J., Roy A. & Zhang Y. Protein-ligand binding site recognition using complementary binding-specific substructure comparison and sequence profile alignment. Bioinformatics 29, 2588–2595 (2013). 23975762 10.1093/bioinformatics/btt447 PMC3789548

[b35] Huang F. & Mazin A. V. A small molecule inhibitor of human RAD51 potentiates breast cancer cell killing by therapeutic agents in mouse xenografts. PLoS One 9, e100993 (2014). 24971740 10.1371/journal.pone.0100993 PMC4074124

[b36] Huang F. & Mazin A. V. Targeting the homologous recombination pathway by small molecule modulators. Bioorg Med Chem Lett 24, 3006–3013 (2014). 24856061 10.1016/j.bmcl.2014.04.088 PMC5568029

[b37] Alagpulinsa D. A., Ayyadevara S. & Shmookler Reis R. J. A Small-Molecule Inhibitor of RAD51 Reduces Homologous Recombination and Sensitizes Multiple Myeloma Cells to Doxorubicin. Front Oncol 4, 289 (2014). 25401086 10.3389/fonc.2014.00289 PMC4214226

[b38] Alpi A., Pasierbek P., Gartner A. & Loidl J. Genetic and cytological characterization of the recombination protein RAD-51 in Caenorhabditis elegans. Chromosoma 112, 6–16 (2003). 12684824 10.1007/s00412-003-0237-5

[b39] Aouida M., Rubio-Texeira M., Thevelein J. M., Poulin R. & Ramotar D. Agp2, a member of the yeast amino acid permease family, positively regulates polyamine transport at the transcriptional level. PLoS One 8, e65717 (2013). 23755272 10.1371/journal.pone.0065717 PMC3670898

[b40] Popova Y., Thayumanavan P., Lonati E., Agrochao M. & Thevelein J. M. Transport and signaling through the phosphate-binding site of the yeast Pho84 phosphate transceptor. Proc Natl Acad Sci USA 107, 2890–2895 (2010). 20133652 10.1073/pnas.0906546107 PMC2840322

[b41] Schothorst J. *et al.* Yeast nutrient transceptors provide novel insight in the functionality of membrane transporters. Curr Genet 59, 197–206 (2013). 24114446 10.1007/s00294-013-0413-y PMC3824880

[b42] Gaber R. F., Ottow K., Andersen H. A. & Kielland-Brandt M. C. Constitutive and hyperresponsive signaling by mutant forms of Saccharomyces cerevisiae amino acid sensor Ssy1. Eukaryot Cell 2, 922–929 (2003). 14555474 10.1128/EC.2.5.922-929.2003 PMC219377

[b43] Didion T., Regenberg B., Jorgensen M. U., Kielland-Brandt M. C. & Andersen H. A. The permease homologue Ssy1p controls the expression of amino acid and peptide transporter genes in Saccharomyces cerevisiae. Mol Microbiol 27, 643–650 (1998). 9489675 10.1046/j.1365-2958.1998.00714.x

[b44] Ljungdahl P. O. Amino-acid-induced signalling via the SPS-sensing pathway in yeast. Biochem Soc Trans 37, 242–247 (2009). 19143640 10.1042/BST0370242

[b45] Bianchi L. & Diez-Sampedro A. A single amino acid change converts the sugar sensor SGLT3 into a sugar transporter. PLoS One 5, e10241 (2010). 20421923 10.1371/journal.pone.0010241 PMC2857651

[b46] Shu Y. *et al.* Evolutionary conservation predicts function of variants of the human organic cation transporter, OCT1. Proc Natl Acad Sci USA 100, 5902–5907 (2003). 12719534 10.1073/pnas.0730858100 PMC156299

[b47] Zhang S. *et al.* Organic cation transporters are determinants of oxaliplatin cytotoxicity. Cancer Res 66, 8847–8857 (2006). 16951202 10.1158/0008-5472.CAN-06-0769 PMC2775093

[b48] Schneider C. A., Rasband W. S. & Eliceiri K. W. NIH Image to ImageJ: 25 years of image analysis. Nat Methods 9, 671–675 (2012). 22930834 10.1038/nmeth.2089 PMC5554542

[b49] McCloy R. A. *et al.* Partial inhibition of Cdk1 in G 2 phase overrides the SAC and decouples mitotic events. Cell Cycle 13, 1400–1412 (2014). 24626186 10.4161/cc.28401 PMC4050138

[b50] Kamath R. S. & Ahringer J. Genome-wide RNAi screening in Caenorhabditis elegans. Methods 30, 313–321 (2003). 12828945 10.1016/s1046-2023(03)00050-1

[b51] Yang J. S. *et al.* OASIS: online application for the survival analysis of lifespan assays performed in aging research. PLoS One 6, e23525 (2011). 21858155 10.1371/journal.pone.0023525 PMC3156233

[b52] Zhang Y. & Skolnick J. Scoring function for automated assessment of protein structure template quality. Proteins 57, 702–710 (2004). 15476259 10.1002/prot.20264

[b53] Xu D. & Zhang Y. Improving the physical realism and structural accuracy of protein models by a two-step atomic-level energy minimization. Biophys J 101, 2525–2534 (2011). 22098752 10.1016/j.bpj.2011.10.024 PMC3218324

[b54] Colubri A. *et al.* Minimalist representations and the importance of nearest neighbor effects in protein folding simulations. J Mol Biol 363, 835–857 (2006). 16982067 10.1016/j.jmb.2006.08.035

[b55] Pei J. & Grishin N. V. PROMALS3D: multiple protein sequence alignment enhanced with evolutionary and three-dimensional structural information. Methods Mol Biol 1079, 263–271 (2014). 24170408 10.1007/978-1-62703-646-7_17 PMC4506754

[b56] Yang J., Wang Y. & Zhang Y. ResQ: An Approach to Unified Estimation of B-Factor and Residue-Specific Error in Protein Structure Prediction. J Mol Biol (2015). 10.1016/j.jmb.2015.09.024 PMC4783266 26437129

[b57] Lomize M. A., Pogozheva I. D., Joo H., Mosberg H. I. & Lomize A. L. OPM database and PPM web server: resources for positioning of proteins in membranes. Nucleic Acids Res 40, D370–D376 (2012). 21890895 10.1093/nar/gkr703 PMC3245162

[b58] von Heijne G. Membrane protein structure prediction. Hydrophobicity analysis and the positive-inside rule. J Mol Biol 225, 487–494 (1992). 1593632 10.1016/0022-2836(92)90934-c

[b59] Omasits U., Ahrens C. H., Muller S. & Wollscheid B. Protter: interactive protein feature visualization and integration with experimental proteomic data. Bioinformatics 30, 884–886 (2014). 24162465 10.1093/bioinformatics/btt607

[b60] Kim S. *et al.* PubChem Substance and Compound databases. Nucleic Acids Res (2015). 10.1093/nar/gkv951 PMC4702940 26400175

