# Supplementary material for: A novel approach using C. elegans DNA damage-induced apoptosis to characterize the dynamics of uptake transporters for therapeutic drug discoveries
Source: Sci Rep. 2016 Oct 27;6:36026. doi: 10.1038/srep36026 (PMC5081529; doi:10.1038/srep36026)
Supplement: Supplementary Table 1 [file srep36026-s1.doc]

**SUPPLEMENTAL DATA**

**Title: A novel approach using *C. elegans* DNA damage-induced apoptosis to characterize the dynamics of uptake transporters for therapeutic drug discoveries**

Arturo Papaluca and Dindial Ramotar

| **Table 1:** *In vivo* validation of ligand-protein docking analysis | | | | | | | |
| --- | --- | --- | --- | --- | --- | --- | --- |
|  |  | Docking Score | | DNA Repair Pathways / Apoptotic cells counts | | | |
| Ligand | | OCT-1 | OCT-2 | HR *- rad-51(ok2218);*  *oct-1(RNAi)* | BER *- apn-1(tm6691);*  *oct-1(RNAi)* | NER *- xpa-1(ok698);*  *oct-1(RNAi)* | MMR *- msh-2(ok2410);*  *oct-1(RNAi)* |
| B02 ‘α’ | 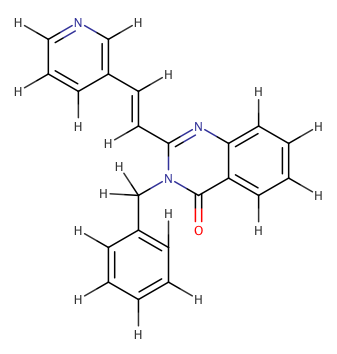 | 0.0 | 4.5 | >20 | >20 | 15-20 | 15-20 |
| Camptothecin | 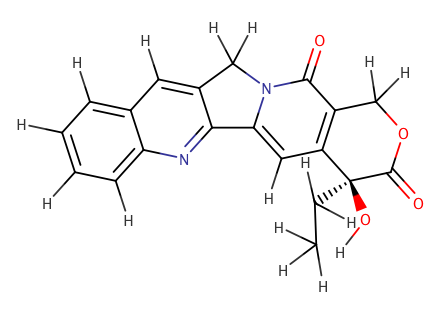 | 3.2 | 3.4 | 15-20 | 15-20 | <5 | <5 |
| Cisplatin | 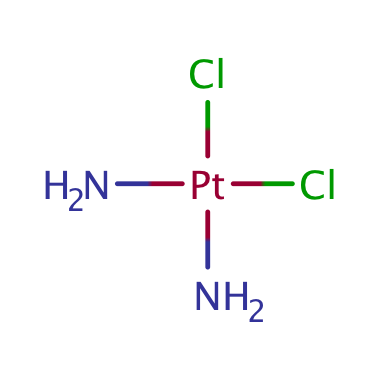 | 3.4 | 4.5 | 5-10 | 5-10 | 15-20 | 15-20 |
| Cycloheximide | 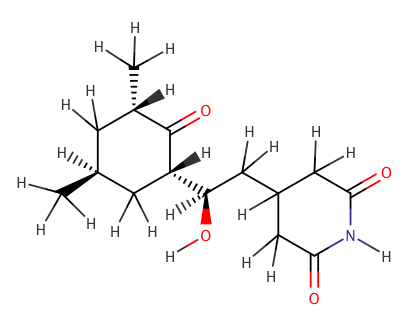 | 6.5 | 7.7 | 15-20 | <5 | <5 | <5 |
| Diclofenac ‘Φ’ | 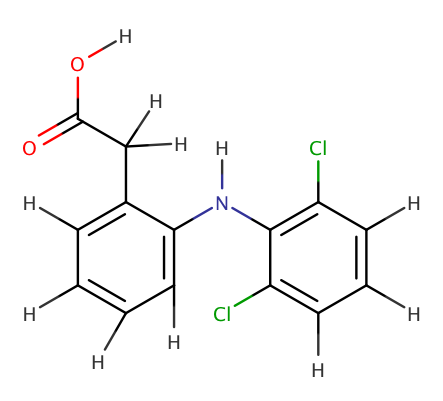 | 0.0 | 0.0 | N.D | N.D | N.D | N.D |
| Doxorubicin | 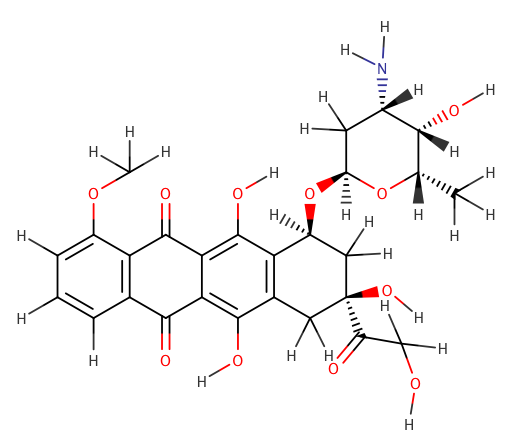 | 3.8 | 4.0 | >20 | 15-20 | <5 | <5 |
| Ketamine ‘Φ’ | 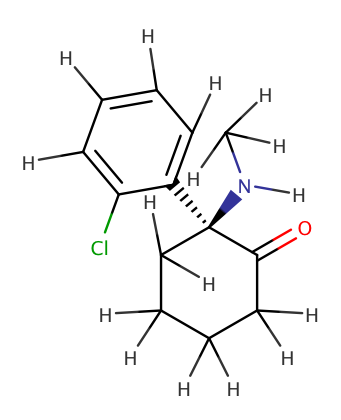 | 4.6 | 7.6 | N.D | N.D | N.D | N.D |
| Melphalan ‘Ω’ | 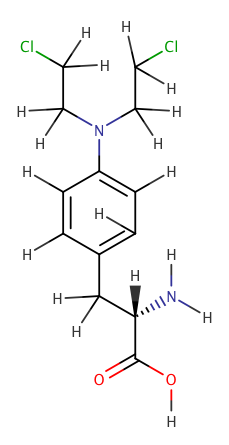 | 0.0 | 0.0 | <5 | 15-20 | <5 | <5 |
| Metformin | 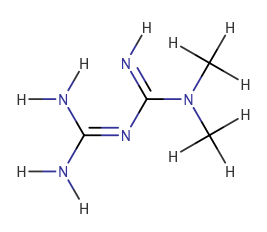 | 0.0 | 3.6 | N.D | N.D | N.D | N.D |
| Methotrexate | 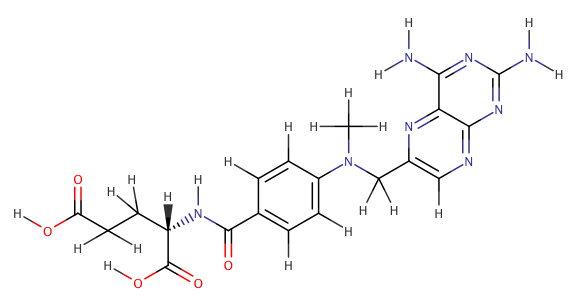 | 5.1 | 7.3 | <5 | 15-20 | <5 | 15-20 |
| Methoxyamine ‘Ω’ | 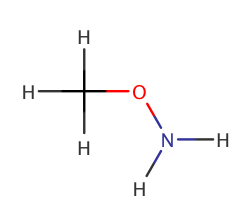 | 0.0 | 0.0 | <5 | >20 | <5 | <5 |
| Methyl metanesulfonate ‘Ω’ | 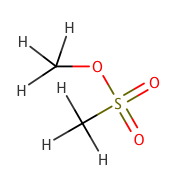 | 0.0 | 0.0 | >20 | 15-20 | 5-10 | <5 |
| Nicotinamide | 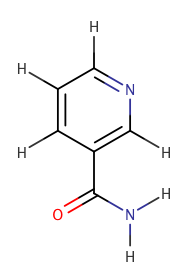 | 0.0 | 5.5 | <5 | >20 | 15-20 | 5-10 |
| 4-Nitroquinoline N-oxide | 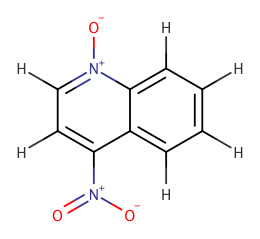 | 2.3 | 4.2 | <5 | <5 | 15-20 | <5 |
| Olaparib ‘Φ’ | 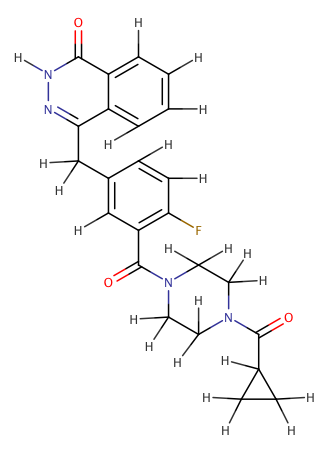 | 5.4 | 6.1 | N.D | N.D | N.D | N.D |
| Paraquat | 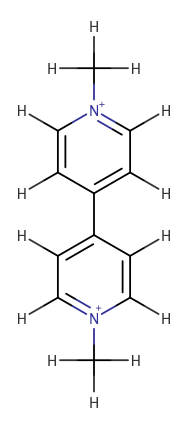 | 3.1 | 3.4 | <5 | >20 | <5 | <5 |
| Phenformin | 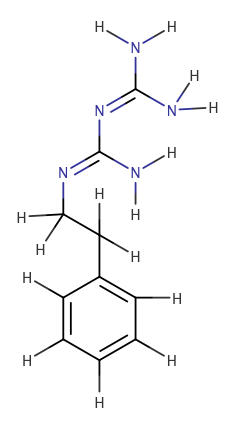 | 7.7 | 7.2 | <5 | <5 | <5 | <5 |
| Puromycin | 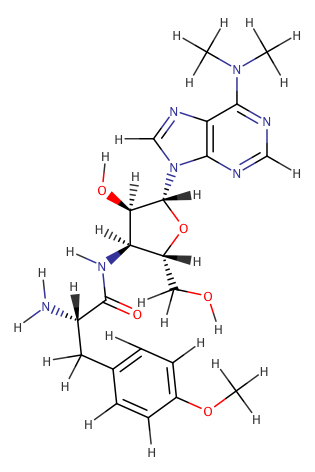 | 5.3 | 7.0 | <5 | 15-20 | <5 | <5 |
| Zeocin | 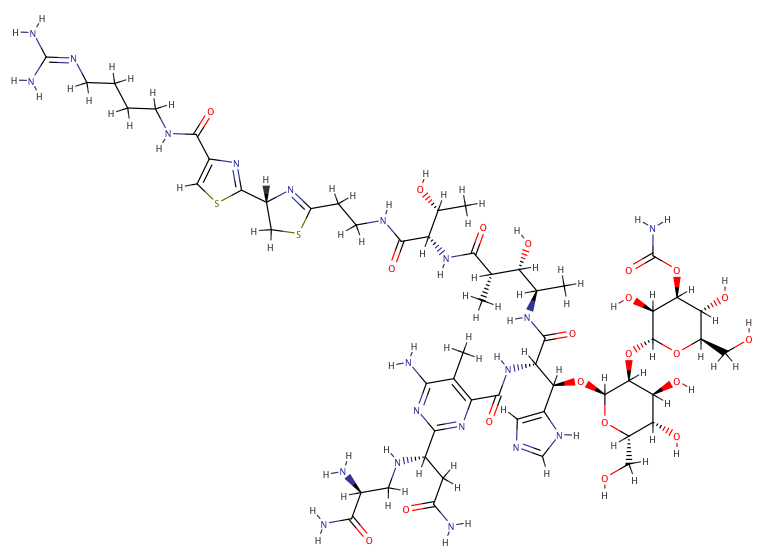 | 8.5 | 6.1 | >20 | <5 | <5 | <5 |

See Table S6 for detailed mechanism of action of each drug used in this assay.

Ligand-protein docking scores: > 3 ; binder | 0 ; non-binder

An average of 2 – 5 apoptotic cells were counted in not treated wild type and *“mutant”; oct-2(RNAi)* animals. After exposure to the drugs, wild type animals depicted an average of 7 – 12 apoptotic cells, whereas *“mutant”; oct-2(RNAi)* animals were comparable to the not treated animals.

‘α’ Wild type animals treated with 5 μM B02 RAD51 inhibitor, signal similar apoptosis-induced response as the drug-induced *rad-51(ok1051)* deletion mutant. B02 caused the animals to become sterile and/or animals that lay eggs that do not hatch as the *rad-51* homozygous sterile deletion (Rinaldo *et al.* 2002) (Figure 5c)

‘Φ’ Predicted virtually

‘Ω’ Uptake by a different transporter

‘N.D’ Not Determined

Chemical structures were rendered utilizing MarvinView 16.1.18 (ChemAxon)
