# Supplementary material for: A novel approach using C. elegans DNA damage-induced apoptosis to characterize the dynamics of uptake transporters for therapeutic drug discoveries
Source: Sci Rep. 2016 Oct 27;6:36026. doi: 10.1038/srep36026 (PMC5081529; doi:10.1038/srep36026)
Supplement: Supplementary Information [file srep36026-s2.pdf]

Supplemental information
Title: A novel approach using *C. elegans* DNA damage-induced apoptosis to characterize the dynamics of uptake transporters for therapeutic drug discoveries

Arturo Papaluca and Dindial Ramotar

Table S1a: Threading template structures used for predicting *C. elegans* OCT-1

Rank[a]	PDB Hit[b]	Protein name[c]	Specie[d]	Identity 1[e]	Identity 2[e]	Normalized Z-Score[f]	Method[g]
1	5c65A	GLUT3 / SLC2A3	*Homo sapiens*	0.18	0.20	1.48	X-Ray diffraction
2	4gbyA	MFS (major facilitator superfamily) proton:xylose symporter XylE	*Escherichia coli*	0.20	0.17	3.09	X-Ray diffraction
3	5c65A	GLUT3 / SLC2A3	*Homo sapiens*	0.18	0.20	2.38	X-Ray diffraction
4	4pypA	GLUT1	*Homo sapiens*	0.18	0.19	3.51	X-Ray diffraction
5	4pypA	GLUT1	*Homo sapiens*	0.18	0.19	2.86	X-Ray diffraction
6	5c65A	GLUT3 / SLC2A3	*Homo sapiens*	0.17	0.20	2.16	X-Ray diffraction
7	4gc0A	MFS (major facilitator superfamily) proton:xylose symporter XylE	*Escherichia coli*	0.18	0.17	4.17	X-Ray diffraction
8	4gc0A	MFS (major facilitator superfamily) proton:xylose symporter XylE	*Escherichia coli*	0.18	0.17	3.40	X-Ray diffraction
9	4ldsA	Inward-facing structure of glucose transporter	*Staphylococcus epidermidis*	0.21	0.18	1.44	X-Ray diffraction
10	4ldsA	Inward-facing structure of glucose transporter	*Staphylococcus epidermidis*	0.21	0.18	1.31	X-Ray diffraction

Table S1b: Threading template structures used for predicting *C. elegans* OCT-2

Rank[a]	PDB Hit[b]	Protein name[c]	Specie[d]	Identity 1[e]	Identity 2[e]	Normalized Z-Score[f]	Method[g]
1	5c65A	GLUT3 / SLC2A3	*Homo sapiens*	0.18	0.16	1.53	X-Ray diffraction
2	4gbyA	MFS (major facilitator superfamily) proton:xylose	*Homo sapiens*	0.18	0.18	2.92	X-Ray diffraction

		symporter XylE					
3	5c65A	GLUT3 / SLC2A3	*Escherichia coli*	0.17	0.16	2.38	X-Ray diffraction
4	4pypA	GLUT1	*Homo sapiens*	0.18	0.16	3.46	X-Ray diffraction
5	4gc0A	MFS (major facilitator superfamily) proton:xylose symporter XylE	*Escherichia coli*	0.18	0.18	2.86	X-Ray diffraction
6	5c65A	GLUT3 / SLC2A3	*Escherichia coli*	0.16	0.16	2.18	X-Ray diffraction
7	4gc0A	MFS (major facilitator superfamily) proton:xylose symporter XylE	*Escherichia coli*	0.17	0.18	4.21	X-Ray diffraction
8	4gc0A	MFS (major facilitator superfamily) proton:xylose symporter XylE	*Escherichia coli*	0.19	0.18	4.28	X-Ray diffraction
9	5c65A	GLUT3 / SLC2A3	*Escherichia coli*	0.18	0.16	1.51	X-Ray diffraction
10	4ldsA	Glucose transporter	*Staphylococcus epidermidis*	0.22	0.16	2.55	X-Ray diffraction

[a]Rank of templates represents the top ten threading templates used by I-TASSER.
[b]PDB Hit IDs from reported protein structures used as threading templates.
[e]Identity 1 and 2 are the percentage sequence identity of the templates in the threading aligned region with the query sequence.
[f]Normalized Z-score is the normalized Z-score of the threading alignments. Alignment with a Z-score >1 signifies a correct alignment.
[g]Method used to resolved the crystal structure.
*The top 10 alignments reported above (in order of their ranking) are from the following threading programs: 1: MUSTER 2: FFAS-3D 3: SPARKS-X 4: HHSEARCH2 5: HHSEARCH I 6: Neff-PPAS 7: HHSEARCH 8: pGenTHREADER 9: wdPPAS 10: cdPPAS.

Table S2a: Top 10 identified structural analogs in PDB database for OCT-1

Rank[a]	PDB Hit[b]	TM-score[c]	RMSD[d]	Identity[e]	Coverage[f]
1	5c65A	0.713	2.15	0.172	0.756
2	4pypA	0.689	2.00	0.184	0.723
3	4gbyA	0.675	2.58	0.147	0.732
4	4ldsA	0.628	2.70	0.190	0.685
5	4j05A	0.583	3.18	0.194	0.644
6	3wdoA	0.573	3.79	0.106	0.665
7	4zowA	0.560	3.35	0.131	0.632
8	1pw4A	0.553	4.17	0.102	0.663
9	4w6vA	0.552	4.23	0.076	0.658
10	4ikvA	0.546	4.23	0.072	0.655

Table S2b: Top 10 identified structural analogs in PDB database for OCT-2

Rank[a]	PDB Hit[b]	TM-score[c]	RMSD[d]	Identity[e]	Coverage[f]
1	4gc0A	0.675	1.63	0.182	0.694
2	5c65A	0.670	2.19	0.165	0.707
3	4ybqA	0.643	2.54	0.181	0.686
4	4pypA	0.593	3.54	0.151	0.675
5	4ldsA	0.564	3.62	0.175	0.646
6	3wdoA	0.556	3.43	0.122	0.624
7	4j05A	0.551	3.47	0.163	0.616
8	3o7qA	0.533	3.28	0.115	0.597
9	4zowA	0.518	3.90	0.108	0.603
10	4m64A	0.511	4.46	0.120	0.617

[a]Rank of templates represents the top ten threading templates used by I-TASSER.
[b]PDB Hit IDs from reported protein structures used as threading templates.
[c]TM-score is a metric for measuring the structural similarity of two protein models. It is designed to solve two major problems in the traditional metrics such as root-mean-square deviation (RMSD): (1) TM-score measures the global fold similarity and is less sensitive to the local structural variations; (2) magnitude of TM-score for random structure pairs is length-independent. TM-score has the value in [0,1], where 1 indicates a perfect match between two structures. Following strict statistics of structures in the PDB, scores below 0.17 corresponds to randomly chosen unrelated proteins whereas with a score higher than 0.5 assume generally the same fold in SCOP/CATH.
[d]RMSD is the root-mean-square deviation between residues that are structurally aligned by TM-align.
[e]Identity is the percentage sequence identity in the structurally aligned region.
[f]Coverage represents the coverage of the alignment by TM-align and is equal to the number of structurally aligned residues divided by length of the query protein.

Table S3: Confidence measurement of *C. elegans* OCT-1 and OCT-2 structures computed with I-TASSER, and after structural refinement with ModRefiner and Modeller

	I-TASSER			ModRefiner		Modeller
Protein	C-score[a]	RMSD[b]	TM-score[c]	RMSD[b]	TM-score[d]	Z-DOPE[e]
OCT-1 (F52F12.1)	-2.35	13.4±4.0Å	0.44±0.14	25.78	0.24	-1.765
OCT-2 (ZK455.8)	-2.52	14.1±3.8Å	0.42±0.14	18.03	0.35	-0.472

[a]C-score is a confidence score for estimating the quality of predicted models by I-TASSER. C-score range between [-5 and 2], where a C-score of higher value signifies a model with a high confidence.
[b]RMSD is the root-mean-square deviation between residues that are structurally aligned[1].
TM-score is the metric for measuring the structural similarity of two protein models. [c]TM-score is based on their correlation with I-Tasser's C-score, and ModRefiner [d]TM-score indicates a model of correct topology whose value range between [>0.5 and <0.17].
[e]Z-DOPE is the atomic distance-dependent statistical calculation from samples of native protein structures. Protein structures computed lower than -1, score as native-like structures.

Table S4: Predicted amino acid positions for ligand-protein interaction:

Protein	Residues	C-score[a]
OCT-1	63, 64, 67, 72, 73, 75, 155, 162, 219, 223, 367, 370, 374, 375, 379, 382, 389, 392, 459, 490, 494	0.38

OCT-2	58, 188, 62, 270, 273, 274, 277, 389, 390, 394, 426, 482, 483, 490, 514, 517	0.75

[a]C-score is the confidence score of predicted binding site. Scores falls in between 0-1; where a score close or equal to 1 signifies a reliable prediction. Amino acids in blue are the ones forming polar contacts with doxorubicin (Fig. 4c).

Table S5: Computed ligand-protein docking scores with BSP-SLIM:

Drug	OCT-1		OCT-2	
	Docking score[a]	Predicted amino acids	Docking score[a]	Predicted amino acids
Doxorubicin	3.805	Pro63, Tyr162, Asn370, Asn375	3.993	Asn58, Tyr188, Trp273, Tyr490, Arg514
Diclofenac	0	0	0	0

[a]Docking score is the confidence score of predicted ligand-protein docking. Scores higher than 1 are considered being a reliable docking.

Table S6: Mechanism of action of the drugs used in this study

Ligand	Mechanism of action	PubChem CID
1. B02	RAD51 inhibitor resulting in unrepaired double strand breaks.	5738263
2. Camptothecin	Inhibits the nuclear enzyme DNA Topoisomerase I.	24360
3. Cisplatin	Produce intra and interstrand DNA crosslinks.	441203
4. Cycloheximide	Inhibits elongation during protein synthesis.	6197
5. Diclofenac	Non-steroidal anti-inflammatory agent	3033
6. Doxorubicin	Intercalates between base pairs in the DNA helix, thereby preventing DNA replication and inhibiting protein synthesis. Inhibits Topoisomerase II.	31703
7. Ketamine	Induction of anesthesia	3821
8. Melphalan	Alkylates DNA at the N7 position of guanine and induces DNA interstrand crosslinkages, resulting in the inhibition of DNA and RNA synthesis and cytotoxicity against both dividing and non-dividing tumor cells.	460612
9. Metformin	Decrease hepatic glucose production, mostly through a mild and transient inhibition of the mitochondrial respiratory-chain complex 1.	4091
10. Methotrexate	Binds to and inhibits the enzyme dihydrofolate reductase, resulting in inhibition of purine nucleotide and thymidylate synthesis and, subsequently, inhibition of DNA and RNA syntheses. Induce oxidative DNA damage.	126941
11. Methoxyamine	Binds to apurinic/apyrimidinic (AP) DNA	4113

	damage sites and inhibits base excision repair (BER), which may result in an increase in DNA strand breaks.	
12. Methyl methanesulfonate	Acts as a mutagen by altering and damaging DNA producing distinct types of lesions.	4156
13. Nicotinamide	Acts as a chemo- and radio-sensitizing agent by enhancing tumor blood flow, thereby reducing tumor hypoxia. This agent also inhibits poly(ADP-ribose) polymerases, enzymes involved in the rejoining of DNA strand breaks induced by radiation or chemotherapy.	936
14. 4-Nitroquinoline N-oxide	4-NQO and its metabolite 4-Hydroxyaminoquinolone-1-oxide bind to nucleic acids.	5955
15. Olaparib	Inhibits PARP-mediated repair of single strand DNA breaks; also enhance the cytotoxicity of DNA-damaging agents.	23725625
16. Paraquat	Catalyze the formation of reactive oxygen species (ROS), more specifically, the superoxide free radical. Paraquat will undergo redox cycling in vivo, being reduced by an electron donor such as NADPH, before being oxidized by an electron receptor such as dioxygen to produce superoxide, a major ROS.	15939
17. Phenformin	Hypoglycemic agent closely related to metformin.	8249
18. Puromycin	Acting as an analog of the 3' terminal end of aminoacyl-tRNA, puromycin incorporates itself into a growing polypeptide chain and causes its premature termination, thereby inhibiting protein synthesis and producing oxidative damages.	439530
19. Zeocin	Acts by intercalating into DNA and induces DNA double strand breaks.	71668282

Title: A novel approach using *C. elegans* DNA damage-induced apoptosis to characterize the dynamics of uptake transporters for therapeutic drug discoveries

Arturo Papaluca and Dindial Ramotar

Legends for Supplemental Figures

Figure S1. Sequence alignment of members belonging to the family of organic cation transporters from *C. elegans* (CeOCT-1 and CeOCT-2), *Homo sapiens* (hOCT1 and hOCT2) and *Mus musculus* (mOCT1 and mOCT2). Numbers indicate amino acid positions. Identical or similar amino acid residues amongst the members are shaded in black or gray, respectively. Dashes indicate gaps. The stretch of amino acid residues, PESPRW (consensus in red), is the longest identical region in all six transporters. CeOCT-2 contains the four conserved cysteine residues Cys203, 250, 280 and 302 present in the N-terminus of each member. The OCT2 from the different species lack the conserved cysteine Cys49, Cys27, and Cys27 present in the OCT1 members CeOCT-1, hOCT1 and mOCT1, respectively.

Figure S2. Relative gene expression. (A) *oct-1* and *oct-2* gene expression are downregulated by the *oct-1(RNAi)* and *oct-2(RNAi)*, respectively. (B) *pes-23* gene expression is not affected by either *oct-1(RNAi)* and *oct-2(RNAi).* The RNA expression was measured as described in the experimental procedures.

Figure S3. *oct-1* and *oct-2* gene expression data measured (A) across all developmental stages and (B) hermaphrodite soma and hermaphrodite gonads. The *oct-1* (WBGene00003842) and *oct-2* (WBGene00003843) RNA expression data was extracted from the Wormbase/SPELL database.

Figure S4. OCT-2-dependent doxorubicin uptake into the pharynx of *C. elegans* is not affected by the eating defective *eat-2(ad453)* mutant animals. Experiment is represented by 'fire' look-up images of the pharynx from eating defective *eat-2(ad453)* untreated and doxorubicin treated animals. The respective DIC images are shown in the upper left corner of each panel. Images to the right of each pharynx depict a 3D representation of the doxorubicin (100 μM) treatment signal intensity for the indicated genotypes. Data are representative of experiments performed in duplicates (n = 15). Enlargement of the pharynx is represented by a scale bar = 10 μm. Fluorescence posterior to the pharynx is auto-fluorescence detected from the intestine.

Figure S5. Genetic analysis of doxorubicin- and cisplatin-induced apoptotic cell death. (A) Representative images of wild type*, *cep-1*, *egl-1*, *ced-9*, *ced-4* and *ced-3* mutant animals untreated and RNAi-driven depletion of oct-1 exposed to 100 μM doxorubicin. Apoptotic cells were observed and quantified as described in the experimental procedures. (B) Apoptotic pathway in *C. elegans* (C) Data shown represent the average quantification of three independent experiments (n =30). *Images from Figure 2 were used for comparison purposes.

Figure S6. Methyl methanesulfonate and Gamma rays (γ-rays)-induced germ cell apoptosis are independent of OCT-1 and OCT-2 function. (A and C) Box and whisker plots depict quantification of apoptotic cell corpses upon MMS (0.25 μM) and γ-rays (75 grays) treatment, respectively. (B) Representative images of right gonad arms after exposure to γ-rays. Posterior is right. The results are the averages from three independent

experiments (n = 30) Mann-Whitney U-test (*P<0.05; **P<0.01; ***P<0.001; ****P<0.0001 and N.S. = Non Significant).

Figure S7. RNAi-driven downregulation of *oct-1* upregulates *oct-2* expression and sensitizes *C. elegans* DNA repair deficient mutants to drug-induced apoptotic cell death. (A) Wild type. (B and C) The homologous recombination mutant *rad-51(ok2218)* downregulated for oct-1 shows stimulated doxorubicin-induced apoptotic cell death. (D and E) The base excision repair mutant *apn-1(tm6691)* downregulated for *oct-1* displays enhanced spontaneous, as well as doxorubicin-induced apoptotic cell death. (F and G and H and I) The nucleotide excision and mismatch repair defective mutants, *xpa-1(ok698)* and *msh-2(ok2410)*, respectively, downregulated for *oct-1* exhibit enhanced cisplatin-induced apoptotic cell death. Treatment with doxorubicin (100 µM, red boxes) and cisplatin (100 µM, blue boxes) started with L1-staged animals. Apoptotic cell corpses were analysed in young adult staged animals. Untreated animals are depicted as white boxes. The results are the averages from three independent experiments (n = 30 each). Mann-Whitney U-test of mean difference (*P<0.05; **P<0.01; ***P<0.001 and ****P<0.0001) was calculated. (J - M) RNAi-driven downregulation of *oct-1* upregulates *oct-2* in the wild type and the DNA repair defective mutants. The Y-axis represents the same scale for *oct-1* and *oct-2* gene expression in all genotypes.

Figure S8. Structural modeling prediction of (A) OCT-1 and (B) OCT-2 computed with ResQ. The predicted Normalized B-factor and estimated residues accuracy in Ångstrom

were computed based on the I-TASSER models. The twelve transmembrane domains are represented as red bars at the bottom of each panel.

Figure S9. Apoptotic cell corpses as a function of cisplatin concentrations. At 25 µM of cisplatin, apoptotic cell corpses were induced in the *oct-1(ok1051)* mutant, but not in the wild type.

Title: A novel approach using *C. elegans* DNA damage-induced apoptosis to characterize the dynamics of uptake transporters for therapeutic drug discoveries

Arturo Papaluca and Dindial Ramotar

```
CeOCT-1      1 MSFQAMETFAEISQEILMSATKPP-D-----------------------FDFVLEQVGNY
CeOCT-2      1 M----------------------N-QHYQKQKQYASTKETRAKRLTDIDFEGILQLIGGC
hOCT1        1 M----------------------P-T-----------------------VDDILEQVGES
hOCT2        1 M----------------------PTT-----------------------VDDVLEHGGEF
mOCT1        1 M----------------------P-T-----------------------VDDVLEHVGEF
mOCT2        1 M----------------------P-T-----------------------VDDILEHIGEF
consensus    1 M                      p t                       vddvLehvGef

CeOCT-1     37 GTYQIVFFFIICLPTSLPSAFSAFNIPFVVGNPPHTCHIPEGKEYLRP--L---------
CeOCT-2     38 SYWQIIVYLIISVQQV-PHAMFNLSVVYMMYQPDHWCKIPFFNEESFSAELGYTNYTWDQ
hOCT1       15 GWFQKQAFLILCLLSA-AFAPICVGIVFLGFTPDHHCQSPGVAELSQR--CGWS---PAE
hOCT2       16 HFFQKQMFFLLALLSA-TFAPIYVGIVFLGFTPDHRCRSPGVAELSLR--CGWS---PAE
mOCT1       15 GWFQKQAFLLLCLISA-SLAPIYVGIVFLGFTPDHHCRSPGVAELSQR--CGWS---PAE
mOCT2       15 HLFQKQTFFLLALLSG-AFTPIYVGIVFLGFTPNHHCRSPGVAELSQR--CGWS---PAE
consensus   61 gyfQkq f ilcllsa  fapiyvgivflgftPdHhCrsPgvaElsqr  cgws   pae

CeOCT-1     86 ---------------TNDT-QILSCKQY----NETQINVFRAFTSAPVDTYSDRISLVPC
CeOCT-2     97 VLNSTIAFPRTFNKQRNEL-HHDQCHYFERDYVHIKL---SPW--AQVKDMNATGKINRC
hOCT1       69 ELNYTVPGLGP----AGEA-FLGQCRRYEVDWNQSALSCVDPL--ASLATNRSHLPLGPC
hOCT2       70 ELNYTVPGPGP----AGEA-SPRQCRRYEVDWNQSTFDCVDPL--ASLDTNRSRLPLGPC
mOCT1       69 ELNYTVPGLGS----AGEASFLSQCMKYEVDWNQSTLDCVDPL--SSLAANRSHLPLSPC
mOCT2       69 ELNYTVPGLGS----AGEVSFLSQCMRYEVDWNQSTLDCVDPL--SSLAANRSHLPLSPC
consensus  121 elnytvpglgt    agea fl qCkryevdwnqstldcvdpl  aslatnrshlpl pC

CeOCT-1    126 QNGWDYDNSTYLDSLVTEFNLVCDQQAWIEISTTSFYVGSFIGNCLFGYVADKFGRRRSF
CeOCT-2    151 K-EWEYDTSVMDRTIVTEWNRVCDNNWSRAHVHMSYSLGYLVGCFVGGFISDRYGRKTAI
hOCT1      122 QDGWVYDTP--GSSIVTEFNLVCADSWKLDLFQSCLNAGFLFGSLGVGYFADRFGRKLCL
hOCT2      123 RDGWVYETP--GSSIVTEFNLVCANSWMLDLFQSSVNVGFFIGSMSIGYIADRFGRKLCL
mOCT1      123 EHGWVYDTP--GSSIVTEFNLVCGDAWKVDLFQSCVNLGFFLGSLVVGYIADRFGRKLCL
mOCT2      123 EHGWVYDTP--GSSIVTEFNLVCAHSWMLDLFQSLVNVGFFIGAVGIGYLADRFGRKFCL
consensus  181   gWvYdtp  gssiVTEfNlVCaqsw ldlfqssvnvGffiGsllvGyiaDrfGRklcl

CeOCT-1    186 FVILTVLIVCGTASSFAKDIESFIILRFFTGLAFPALFQIPFIICMEFMGNSGRIFSGLM
CeOCT-2    210 TGFGILTMLFGFLLTYSKEFEIFLVVRFLLAATNEAADLAAYVLCMEVTGTKYRSIVGSL
hOCT1      180 LGTVLVNAVSGVLMAFSPNYMSMLLFRLLQGLVSKGNWMAGYTLITEFVGSGSRRTVAIM
hOCT2      181 LTTVLINAAAGVLMAISPTYTWMLIFRLIQGLVSKAGWLIGYILITEFVGRRYRRTVGIF
mOCT1      181 LVTTLVTSLSGVLTAVAPDYTSMLLFRLLQGMVSKGSWVSGYTLITEFVGSGYRRTTAIL
mOCT2      181 LVTILINAISGVLMAISPNYAWMLVFRFLQGLVSKAGWLIGYILITEFVGLGYRRTVGIC
consensus  241 lvtllvnavsGvlma spdy smlifR lqglvskaawligyilitEfvGtgyRrtvgim

CeOCT-1    246 TSLFFGAAMALLGVVAMFIRRWRQLTFFCNAPFAFYIIYYFFLPESPRWSVSVGKWADAK
CeOCT-2    270 IQAPWACGYAFLALIAYLTKSWTMIHLICVLLHIISLMLLYFLPESPRWLILNNKTKQAE
hOCT1      240 YQMAFTVGLVALTGLAYALPHWRWLQLAVSLPTFLFLLYYWCVPESPRWLLSQKRNTEAI
hOCT2      241 YQVAYTVGLLVLAGVAYALPHWRWLQFTVSLPNFFFLLYYWCIPESPRWLISQNKNAEAM
mOCT1      241 YQVAFTVGLVGLAGVAYAIPDWRWLQLAVSLPTFLFLLYYWFVPESPRWLLSQKRTTQAV
mOCT2      241 YQIAFTVGLLILAGVAYALPNWRWLQFAVTLPNFCFLLYFWCIPESPRWLISQNKNAKAM
consensus  301 yqlaftvglvlLagvAyalprWrwlq avslp fifllyyw lPESPRWlisqknadAi

CeOCT-1    306 KQLKKIAKMNGKSN-VDVDELVDSMKNHQNAAEE-KET--KRSHNVTDLFKTPNLRRKTL
CeOCT-2    330 KIIREACHYNKSRLPSDLGL--------VRHAEKKKWMKHNEKPSYFHLFRSSELRFRNV
hOCT1      300 KIMDHIAQKNGKLPPADLKM--------LSLEED-VTE--KLSPSFADLFRTPRLRKRTF
hOCT2      301 RIIKHIAKKNGKSLPASLQR--------LRLEEE-TGK--KLNPSFLDLVRTPQIRKHTM
mOCT1      301 RIMEQIAQKNRKVPPADLKM--------MCLEED-ASE--RRSPSFADLFRTPSLRKHTL
mOCT2      301 KIIKHIAKKNGKSVPVSLQS--------LTADED-TGM--KLNPSFLDLVRTPQIRKHTL
consensus  361 kiikhiakkNgkslpadl l        l leEd      klspsf dLfrtpnlRkhtl
```

```
CeOCT-1     362 IVTYIWVMNAIIYNGLTLNVSNLP--------VDDYWSFIINGAVELPGYFVVWPLLQCA
CeOCT-2     382 VLFIVWIATALVYYGMVIALSDQSSPGRRVFDGNFFLNNAMAGAIELPTLVF-CVFLLRM
hOCT1       349 ILMYLWFTDSVLYQGLILHMGATS--------GNLYLDFLYSALVEIPGAFIALITIDRV
hOCT2       350 ILMYNWFTSSVLYQGLIMHMGLAG--------DNIYLDFFYSALVEFPAAFMIILTIDRI
mOCT1       350 ILMYLWFSCAVLYQGLIMHVGATG--------ANLYLDFFYSSLVEFPAAFIILVTIDRI
mOCT2       350 ILMYNWFTSSVLYQGLIMHMGLAG--------DNIYLDFFYSALVEFPAAFIIILTIDRI
consensus   421 ilmyiWftt vlYqGlimhmg  g        gnlyldffysalvEfPaafiilvtidri

CeOCT-1     414 GRRWTLAATMIVCGIGCVSAMFMP-DGYPWLVASASFIGKFGVGSGFAVIYIFAGELYPT
CeOCT-2     441 GRKRSQMLVLFGSGLFLLTSVVMVYRKQSTLALIFMLLSKACIQGSFNILYIFTSELNPT
hOCT1       401 GRIYPMAMSNLLAGAACLVMIFIS-PDLHWLNIIIMCVGRMGITIAIQMICLVNAELYPT
hOCT2       402 GRRYPWAASNMVAGAACLASVFIP-GDLQWLKIIISCLGRMGITMAYEIVCLVNAELYPT
mOCT1       402 GRIYPIAASNLVAGAACLLMIFIP-HELHWLNVTLACLGRMGATIVLQMVCLVNAELYPT
mOCT2       402 GRRYPWAVSNMVAGAACLASVFIP-DDLQWLKITVACLGRMGITIAYEMVCLVNAELYPT
consensus   481 GRryplaasnivaGaacl svfip  dl wL iii clgrmgitiafnmvclvnaELyPT

CeOCT-1     473 VVRAIGMGMSSMVAGSGLLLAPHIV-NLGKIVK-ILPLLIMGLMALSAGILTFFLPETLG
CeOCT-2     501 VVRNSAVGISSMVARMGAGASGYIA-ILSDVTMPLVPMTIFACFSLLAGCLVLLLPETQG
hOCT1       460 FVRNLGVMVCSSLCDIGGIITPFIVFRLREVWQ-ALPLILFAVLGLLAAGVTLLLPETKG
hOCT2       461 FIRNLGVHICSSMCDIGGIITPFLVYRLTNIWL-ELPLMVFGVLGLVAGGLVLLLPETKG
mOCT1       461 FIRNLGMMVCSALCDLGGIFTPFMVFRLMEVWQ-ALPLILFGVLGLSAGAVTLLLPETKG
mOCT2       461 YIRNLAVLVCSSMCDIGGIVTPFLVYRLTDIWL-EFPLVVFAVVGLVAGGLVLLLPETKG
consensus   541 fvRnlgvmvcSsvcdiGgiltpfivfrLsdiwm  lPllifgvlgLlAggl llLPETkG

CeOCT-1     531 APLPMTIEDAENFGKK-PEPDSG--MFTQ-AAKKRESQPL--LE----------------
CeOCT-2     560 LPLPDTILDSVQMVKRNTKPCGTLSGTFLGGIDDDA-QPYGGKLPPRVSSDDEEEEEEED
hOCT1       519 VALPETMKDAENLGRK-AKPKEN--TIYL-KVQTSE-------P----------------
hOCT2       520 KALPETIEEAENMQRP-RKNKEK--MIYL-QVQKLD-------I----------------
mOCT1       520 VALPETIEEAENLGRRKSKAKEN--TIYL-QVQTGK-------S----------------
mOCT2       520 KALPETIEDAEKMQRP-RKKKEK--RIYL-QVKKAE-------L----------------
consensus   601 laLPeTiedaenmgrk  kpke    iyl qvqk e       l

CeOCT-1     569 -----PHTPMDRRRRSSRLMNI
CeOCT-2     619 SEESIPEKTA------------
hOCT1       552 -----SGT--------------
hOCT2       553 -----PLN--------------
mOCT1       554 -----PHT--------------
mOCT2       553 -------S--------------
consensus   661      p t
```

Figure S1. Sequence alignment of members belonging to the family of organic cation transporters from *C. elegans* (CeOCT-1 and CeOCT-2), *Homo sapiens* (hOCT1 and hOCT2) and *Mus musculus* (mOCT1 and mOCT2). Numbers indicate amino acid positions. Identical or similar amino acid residues amongst the members are shaded in black or gray, respectively. Dashes indicate gaps. The stretch of amino acid residues, PESPRW (consensus in red), is the longest identical region in all six transporters. CeOCT-2 contains the four conserved cysteine residues Cys203, 250, 280 and 302 present in the N-terminus of each member. The OCT2 from the different species lack the conserved cysteine Cys49, Cys27, and Cys27 present in the OCT1 members CeOCT-1, hOCT1 and mOCT1, respectively.

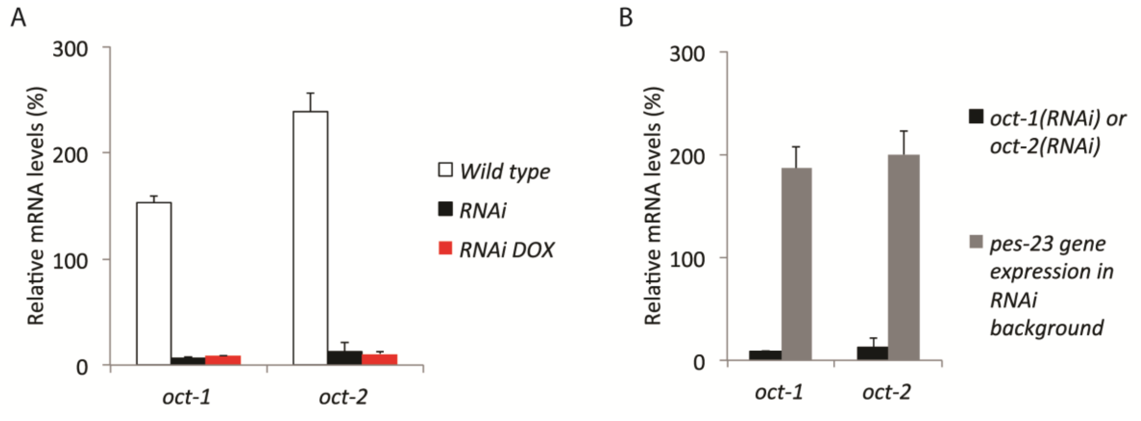

Figure S2. Relative gene expression. (A) *oct-1* and *oct-2* gene expression are downregulated by the *oct-1(RNAi)* and *oct-2(RNAi)*, respectively. (B) *pes-23* gene expression is not affected by either *oct-1(RNAi)* and *oct-2(RNAi).* The RNA expression was measured as described in the experimental procedures.

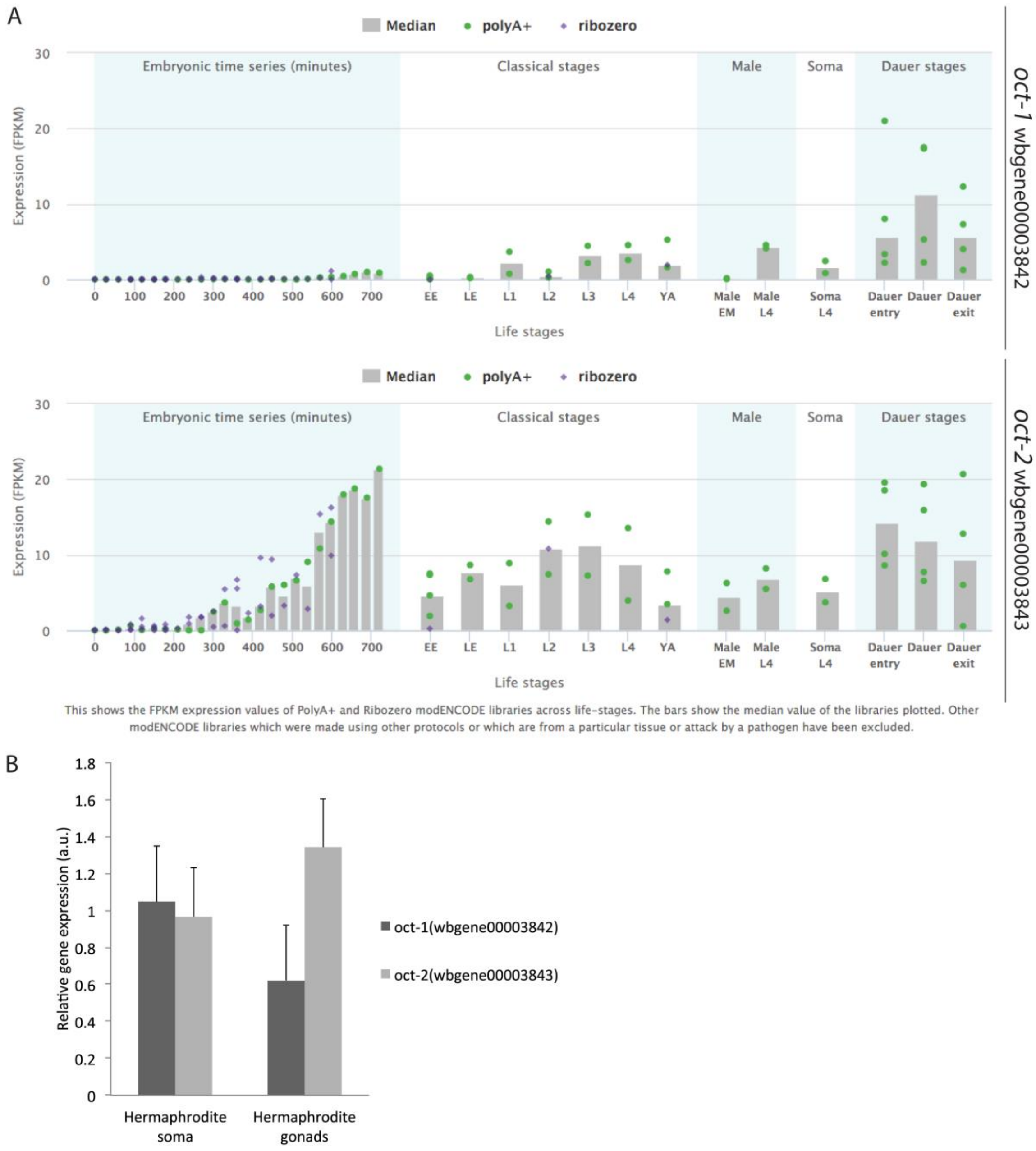

Figure S3. *oct-1* and *oct-2* gene expression data measured (A) across all developmental stages and (B) hermaphrodite soma and hermaphrodite gonads. The *oct-1* (WBGene00003842) and *oct-2* (WBGene00003843) RNA expression data was extracted from the Wormbase/SPELL database.

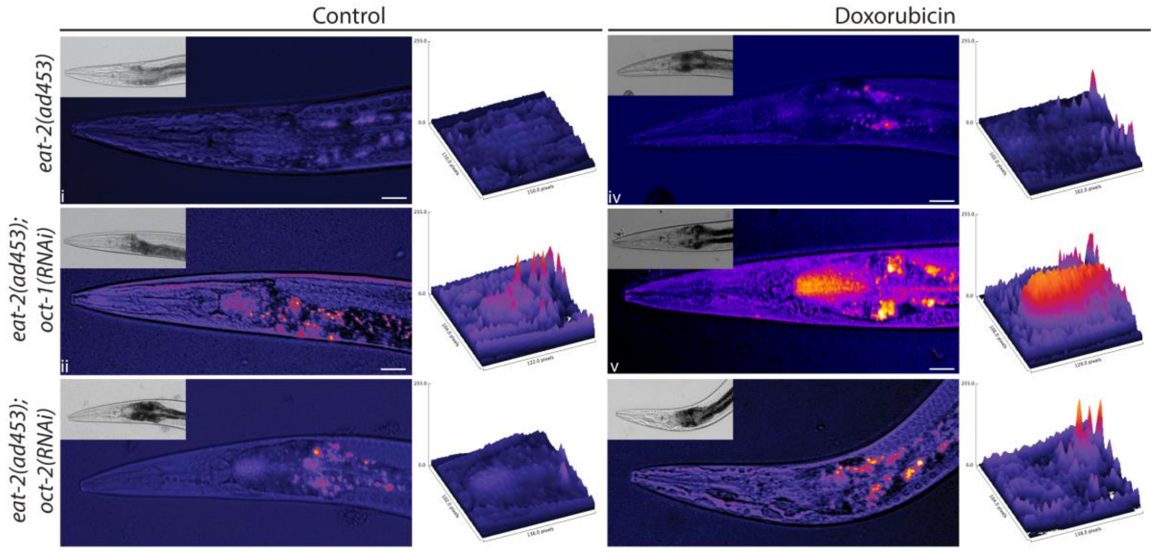

Figure S4. OCT-2-dependent doxorubicin uptake into the pharynx of *C. elegans* is not affected by the eating defective *eat-2(ad453)* mutant animals. Experiment is represented by 'fire' look-up images of the pharynx from eating defective *eat-2(ad453)* untreated and doxorubicin treated animals. The respective DIC images are shown in the upper left corner of each panel. Images to the right of each pharynx depict a 3D representation of the doxorubicin (100 μM) treatment signal intensity for the indicated genotypes. Data are representative of experiments performed in duplicates (n = 15). Enlargement of the pharynx is represented by a scale bar = 10 μm. Fluorescence posterior to the pharynx is auto-fluorescence detected from the intestine.

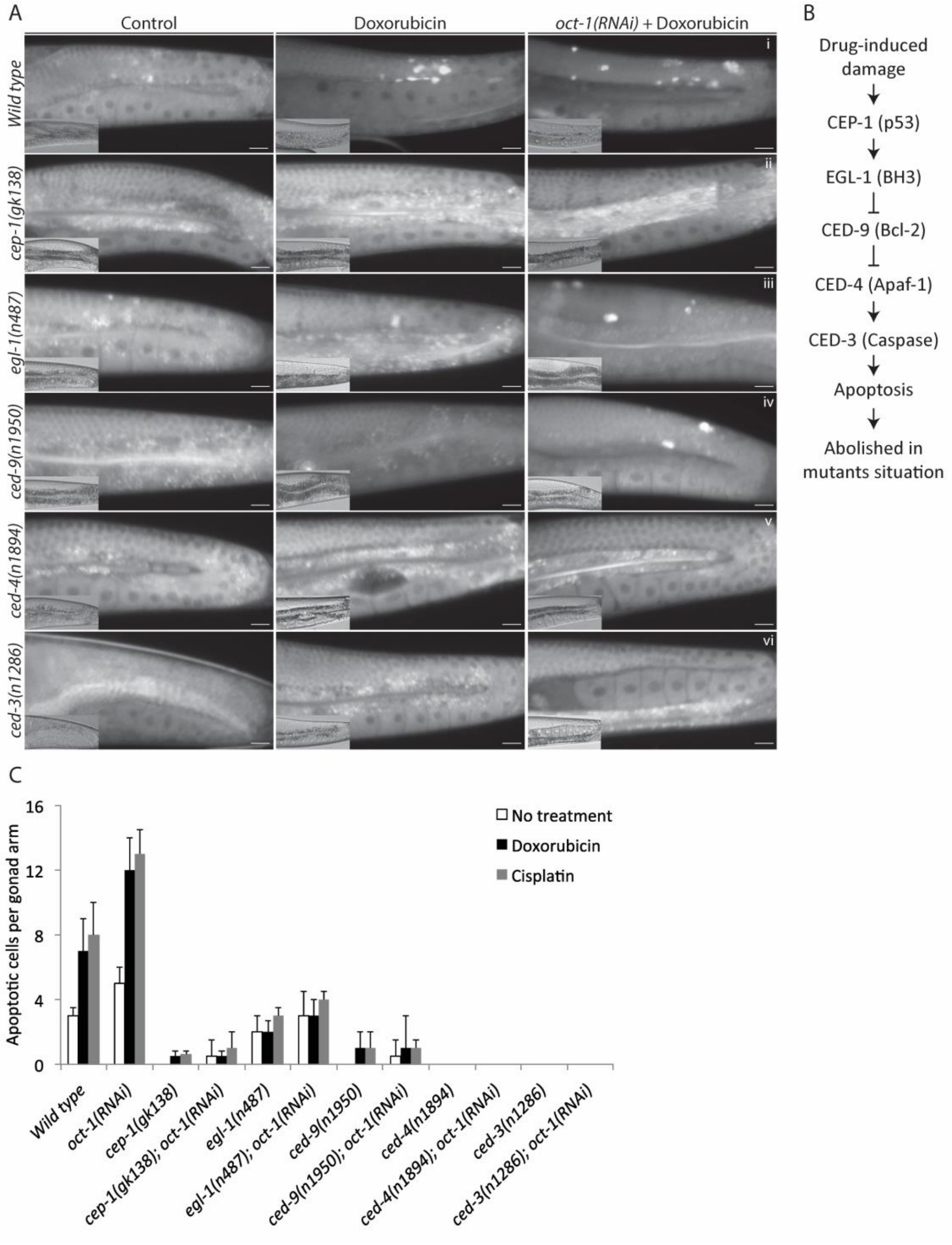

Figure S5. Genetic analysis of doxorubicin- and cisplatin-induced apoptotic cell death. (A) Representative images of wild type*, *cep-1*, *egl-1*, *ced-9*, *ced-4* and *ced-3* mutant animals untreated and RNAi-driven depletion of oct-1 exposed to 100 μM doxorubicin. Apoptotic cells were observed and quantified as described in the experimental procedures. (B) Apoptotic pathway in *C. elegans* (C) Data shown represent the average quantification of three independent experiments (n =30). *Images from Figure 2 were used for comparison purposes.

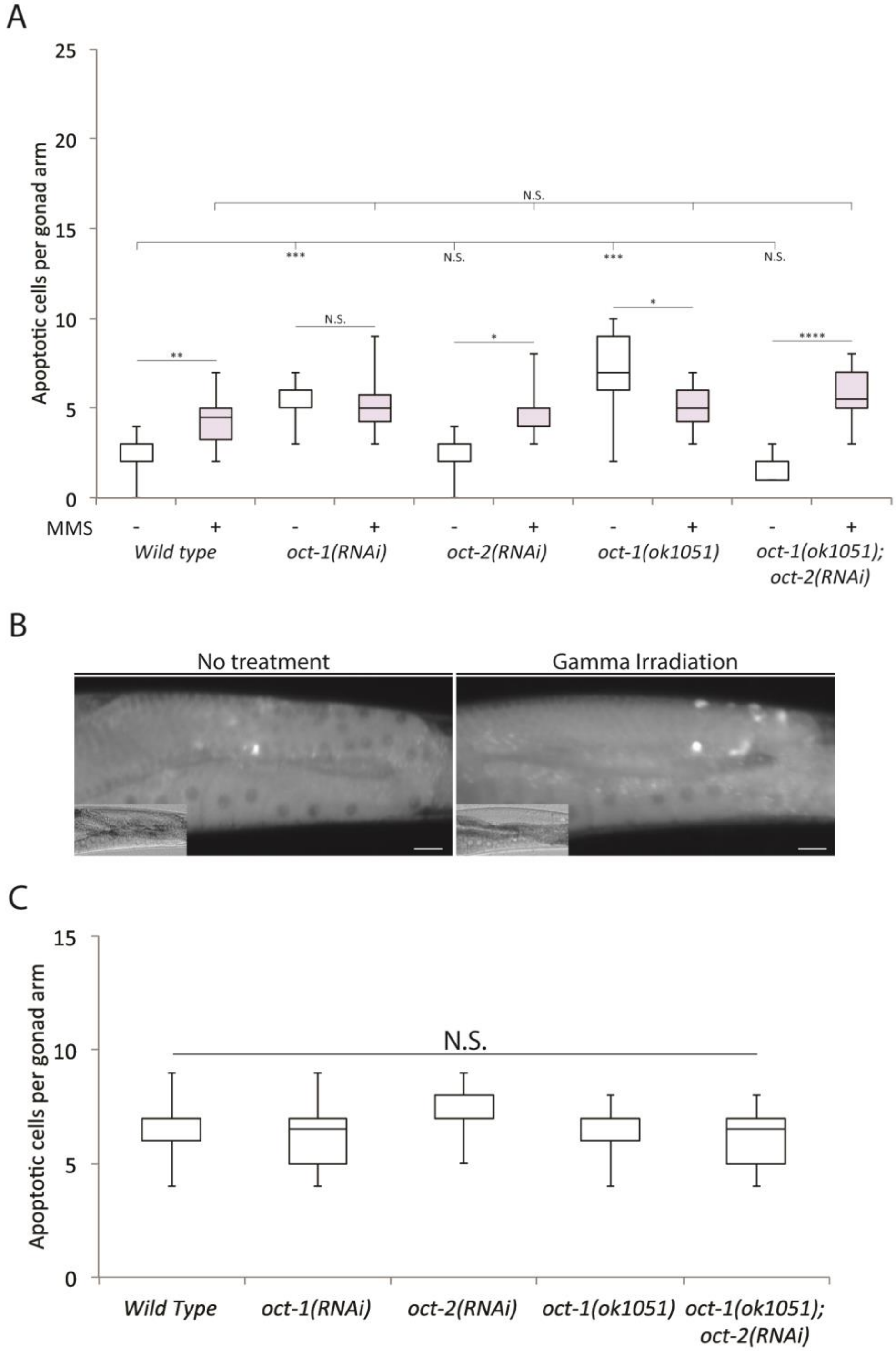

Figure S6. Methyl methanesulfonate and Gamma rays (γ-rays)-induced germ cell apoptosis are independent of OCT-1 and OCT-2 function. (A and C) Box and whisker plots depict quantification of apoptotic cell corpses upon MMS (0.25 μM) and γ-rays (75 grays) treatment, respectively. (B) Representative images of right gonad arms after exposure to γ-rays. Posterior is right. The results are the averages from three independent experiments (n = 30) Mann-Whitney U-test (*P<0.05; **P<0.01; ***P<0.001; ****P<0.0001 and N.S. = Non Significant).

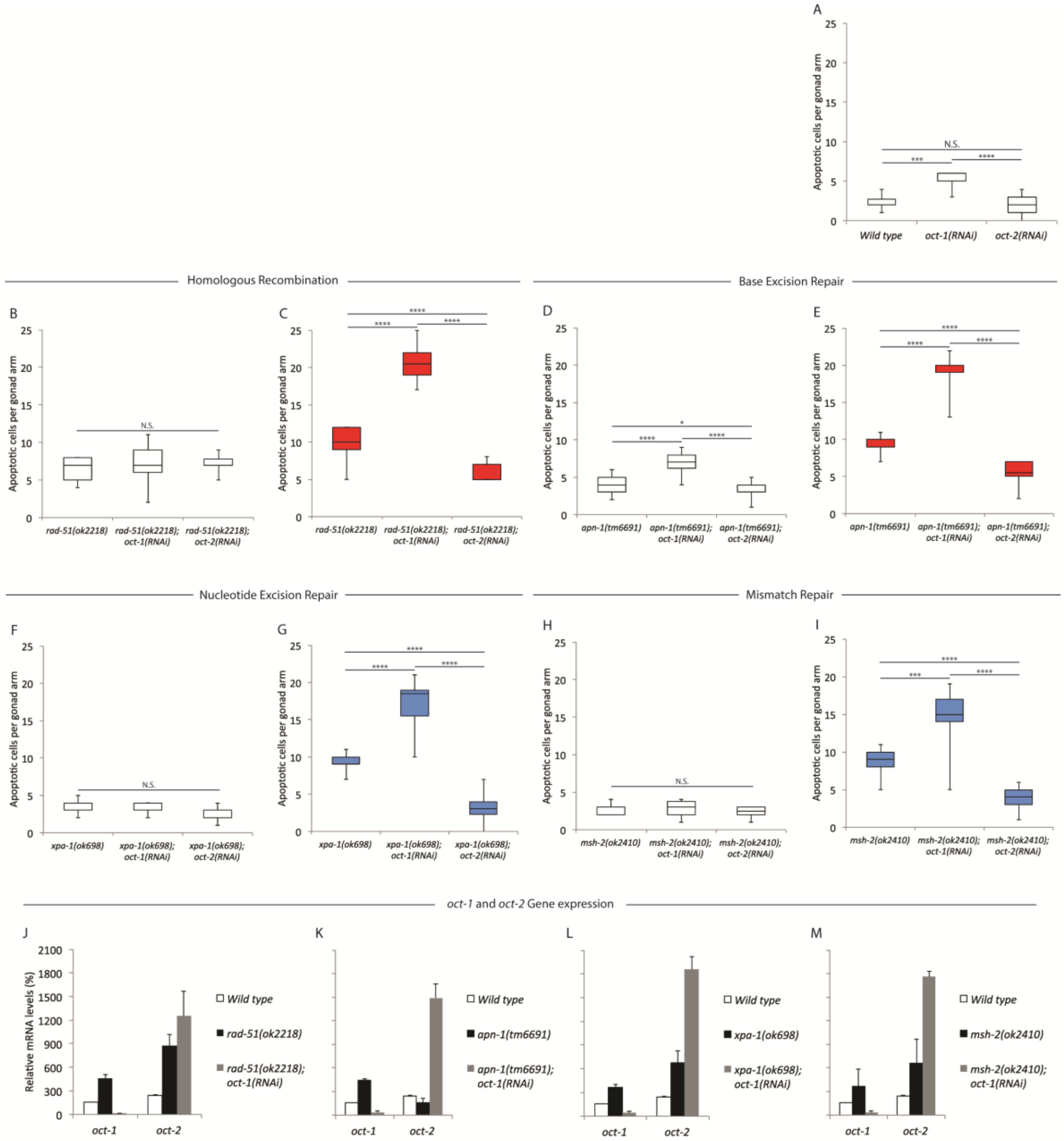

Figure S7. RNAi-driven downregulation of *oct-1* upregulates *oct-2* expression and sensitizes *C. elegans* DNA repair deficient mutants to drug-induced apoptotic cell death. (A) Wild type. (B and C) The homologous recombination mutant *rad-51(ok2218)* downregulated for oct-1 shows stimulated doxorubicin-induced apoptotic cell death. (D and E) The base excision repair mutant *apn-1(tm6691)* downregulated for *oct-1* displays enhanced spontaneous, as well as doxorubicin-induced apoptotic cell death. (F and G and H and I) The nucleotide excision and mismatch repair defective mutants, *xpa-1(ok698)* and *msh-2(ok2410)*, respectively, downregulated for *oct-1* exhibit enhanced cisplatin-induced apoptotic cell death. Treatment with doxorubicin (100 µM, red boxes) and

cisplatin (100 μM, blue boxes) started with L1-staged animals. Apoptotic cell corpses were analysed in young adult staged animals. Untreated animals are depicted as white boxes. The results are the averages from three independent experiments (n = 30 each). Mann-Whitney U-test of mean difference (*P<0.05; **P<0.01; ***P<0.001 and ****P<0.0001) was calculated. (J - M) RNAi-driven downregulation of *oct-1* upregulates *oct-2* in the wild type and the DNA repair defective mutants. The Y-axis represents the same scale for *oct-1* and *oct-2* gene expression in all genotypes.

A: OCT-1

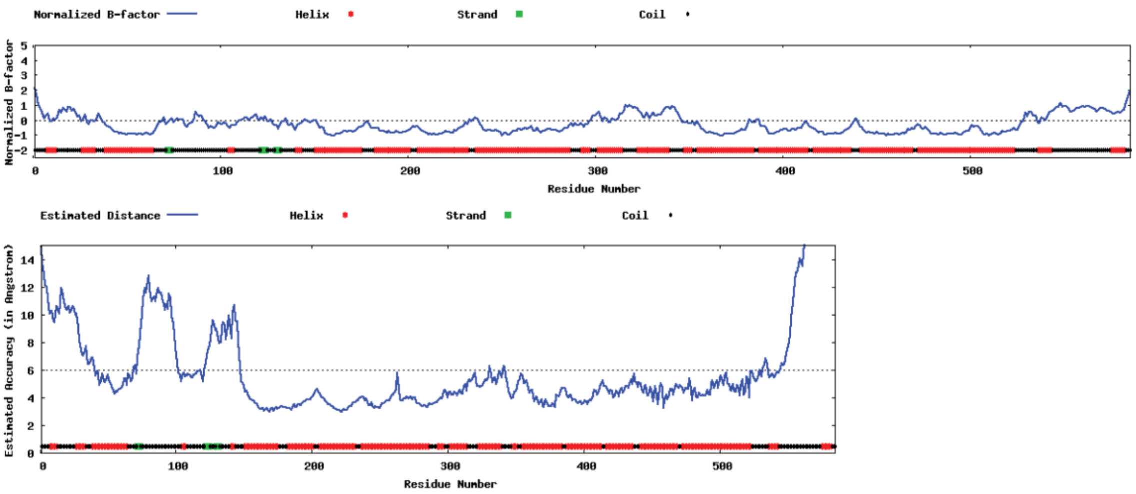

B: OCT-2

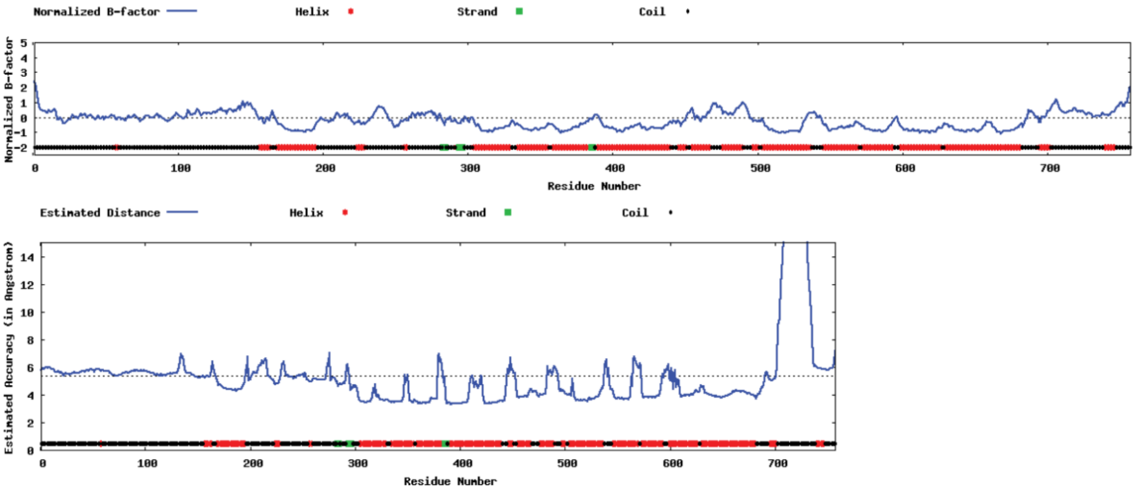

Figure S8. Structural modeling prediction of (A) OCT-1 and (B) OCT-2 computed with ResQ. The predicted Normalized B-factor and estimated residues accuracy in Ångstrom were computed based on the I-TASSER models. The twelve transmembrane domains are represented as red bars at the bottom of each panel.

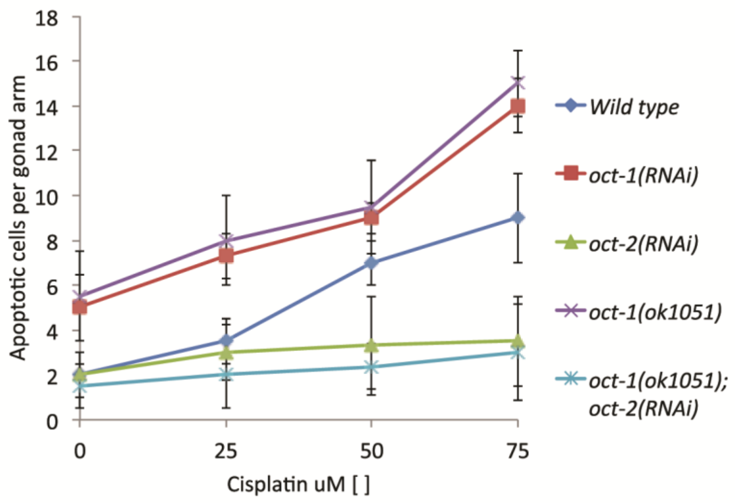

Figure S9. Apoptotic cell corpses as a function of cisplatin concentrations. At 25 µM of cisplatin, apoptotic cell corpses were induced in the *oct-1(ok1051)* mutant, but not in the wild type.
